# *Drosophila* DJ-1 Decreases Neural Sensitivity to Stress by Negatively Regulating Daxx-Like Protein through dFOXO

**DOI:** 10.1371/journal.pgen.1003412

**Published:** 2013-04-04

**Authors:** Soojin Hwang, Saera Song, Yoon Ki Hong, Gahee Choi, Yoon Seok Suh, Seung Yeop Han, Minjung Lee, Seung Hwan Park, Jang Ho Lee, Soojin Lee, Se Min Bang, Yuji Jeong, Won-Ju Chung, Im-Soon Lee, Gilsang Jeong, Jongkyeong Chung, Kyoung Sang Cho

**Affiliations:** 1 Department of Biological Sciences, Konkuk University, Seoul, Republic of Korea; 2 National Creative Research Initiatives Center for Energy Homeostasis Regulation and School of Biological Sciences, Seoul National University, Seoul, Republic of Korea; 3 Laboratory of Environmental Entomology, Department of Agricultural Biology, National Academy of Agricultural Science, Rural Development Administration, Suwon, Republic of Korea; Stanford University School of Medicine, United States of America

## Abstract

*DJ-1*, a Parkinson's disease (PD)–associated gene, has been shown to protect against oxidative stress in *Drosophila*. However, the molecular mechanism underlying oxidative stress-induced phenotypes, including apoptosis, locomotive defects, and lethality, in *DJ-1*-deficient flies is not fully understood. Here we showed that *Daxx-like protein* (*DLP*), a *Drosophila* homologue of the mammalian *Death domain-associated protein* (*Daxx*), was upregulated under oxidative stress conditions in the loss-of-function mutants of *Drosophila DJ-1β*, a *Drosophila* homologue of *DJ-1*. *DLP* overexpression induced apoptosis via the c-Jun N-terminal kinase (JNK)/*Drosophila* forkhead box subgroup O (dFOXO) pathway, whereas loss of *DLP* increased resistance to oxidative stress and UV irradiation. Moreover, the oxidative stress-induced phenotypes of *DJ-1β* mutants were dramatically rescued by *DLP* deficiency, suggesting that enhanced expression of *DLP* contributes to the *DJ-1β* mutant phenotypes. Interestingly, we found that dFOXO was required for the increase in *DLP* expression in *DJ-1β* mutants and that dFOXO activity was increased in the heads of *DJ-1β* mutants. In addition, subcellular localization of DLP appeared to be influenced by *DJ-1* expression so that cytosolic DLP was increased in *DJ-1β* mutants. Similarly, in mammalian cells, Daxx translocation from the nucleus to the cytosol was suppressed by overexpressed *DJ-1β* under oxidative stress conditions; and, furthermore, targeted expression of *DJ-1β* to mitochondria efficiently inhibited the Daxx translocation. Taken together, our findings demonstrate that DJ-1β protects flies against oxidative stress- and UV-induced apoptosis by regulating the subcellular localization and gene expression of DLP, thus implying that Daxx-induced apoptosis is involved in the pathogenesis of *DJ-1*-associated PD.

## Author Summary

DJ-1 is an antioxidant protein that has been implicated in autosomal recessive Parkinson's disease (PD), although the mechanism by which *DJ-1* deficiency causes PD remains elusive. *Drosophila DJ-1* mutants are highly sensitive to oxidative stress and UV irradiation, and oxidative stress-induced cell death is significantly increased in dopaminergic neurons. In this study, we characterized a *Drosophila* homologue of death domain-associated protein (Daxx), *Daxx-like protein* (*DLP*), as a key player in the process of the oxidative stress-induced cell death in *DJ-1* mutants. Upon oxidative stress, *DLP* expression was increased in the *DJ-1* mutants, and locomotive defects and oxidative stress-induced phenotypes including apoptosis and lethality were dramatically rescued by *DLP* deficiency. More interestingly, we revealed that *Drosophila* forkhead box subgroup O was required for the increased *DLP* expression in *DJ-1* mutants. Additionally, *Drosophila* DJ-1 suppressed DLP and Daxx translocation from the nucleus to the cytosol in both fly brain and mammalian cells. Interestingly, targeted expression of *Drosophila DJ-1* to mitochondria efficiently inhibited Daxx translocation. Our results show that *Drosophila* DJ-1 protects dopaminergic neurons from oxidative stresses by regulating the subcellular localization and gene expression of *DLP*, providing a clue to understanding the molecular mechanism underlying oxidative stress-induced neuronal death in PD.

## Introduction

Oxidative stress, a state of imbalance between the generation and elimination of reactive oxygen and nitrogen species, has been implicated in a variety of neurodegenerative diseases [1]–[3]. The central nervous system is presumed to be particularly vulnerable to oxidative stress, as it consumes abundant quantities of oxygen and employs nitric oxide as a biological messenger, both of which create reactive species as by-products [1]. Oxidative stress provokes various cytotoxic processes, such as overstimulation of glutamate receptors (excitotoxicity), ER stress, and mitochondrial dysfunction, which lead to apoptosis, the predominant form of cell death in aging-related neurodegenerative diseases [1], [2].

Parkinson's disease (PD) is characterized by typical motor dysfunction and is thought to be caused by the loss of nigrostriatal dopaminergic (DA) neurons that connect the substantia nigra pars compacta (SNpc) to other brain regions [3]–. The death of these neurons has been closely linked to oxidative stress [3]–[5]. Markers of oxidative damage to lipids, proteins and DNA, as well as mitochondrial DNA deletions, which can be caused by oxidative stress, are significantly elevated in postmortem samples of the SNpc of PD patients [4], [5]. The nigrostriatal pathway is sensitive to 6-hydroxydopamine and 1-methyl-4-phenyl-1,2,3,6-tetrahydropyridine (MPTP)/MPP+, which destroy DA neurons via induction of oxidative stress [6]. Moreover, oxidative stress plays an important role in the function of the familial PD-related genes, *α-synuclein* and *parkin* [3]. Oxidative damaged α-synuclein is aggregated into Lewy bodies [7], and the function of Parkin is impaired by oxidative modifications [8], [9]. Additionally, various animal models of familial PD show greater damage in response to oxidative stress [10]–[16]. Although these data demonstrate a correlation between PD and oxidative stress, the molecular mechanisms underlying oxidative stress-induced DA neuronal death in PD are not well understood.

Among the genes related to PD, *DJ-1* is the most closely associated with oxidative stress [17]. *DJ-1* was originally identified as an oncogene that transforms mouse NIH3T3 cells in cooperation with *ras* [18], and its gene expression is increased in various types of cancer [19]–[21]. Later, *DJ-1* was linked to an autosomal-recessive early-onset type of familial PD [22], [23]. The impairment of DJ-1 function sensitizes animal models to oxidative stress [11], [12], [14], [24]–[26]. DJ-1 performs several critical functions in response to oxidative stress via diverse cellular mechanisms [5], [17], [23]. First, DJ-1 functions as an atypical peroxiredoxin-like peroxidase that scavenges peroxides by oxidizing Cys106 [14]. Second, DJ-1 regulates expression of several antioxidant genes [27]–[30] and stabilizes the antioxidant transcriptional master regulator, Nrf2 [29], [31]. Third, DJ-1 inhibits UV- and oxidative stress-induced cell death by suppressing pro-apoptotic factors [32]–[34]. In *Drosophila*, there are 2 homologues of human *DJ-1*: *DJ-1α* and *β* [11], [12], [24]. *DJ-1α* is predominantly expressed in the testes, whereas *DJ-1β* is expressed in most tissues [11], [24], similar to the expression pattern of mammalian *DJ-1* [18]. Several previous studies have demonstrated that *DJ-1β* loss-of-function mutants are acutely sensitive to oxidative stress and prone to locomotive dysfunction, resembling the phenotypes seen in PD [11], [12], [14], [24].

In this study, we identified a *Drosophila* homologue of death domain-associated protein (Daxx), Daxx-like protein (DLP), as a mediator of *Drosophila DJ-1β* mutant phenotypes. Daxx, originally identified as a binding partner of the pro-apoptotic receptor Fas (also called CD95) [35], performs a pivotal function in apoptosis [36], [37]. Daxx activates apoptosis signal-regulating kinase 1 (ASK1), which in turn increases c-Jun N-terminal kinase (JNK) activity leading to apoptosis [38], [39]. Daxx is increased in cells upon exposure to hydrogen peroxide and functions as a mediator of oxidative stress-induced apoptosis [33], [40], [41]. In neuronal cells, dominant negative-Daxx blocks Fas-induced cell death [42]. In addition, FADD/caspase-8 cascade-triggered cell death requires the transcriptional activation of *Daxx* in normal embryonic motor neurons [43]. Furthermore, Daxx has been identified as a potential component of the pathogenesis of neurodegenerative diseases, including PD [33], [44]. For example, Daxx interacts with DJ-1 [33], and MPTP induces translocation of Daxx from the nucleus to the cytoplasm and activates the ASK1 signaling pathway in mouse SNpc [44].

Although previous studies have demonstrated that *Drosophila DJ-1β* mutants are acutely sensitive to oxidative stress [11], [12], [14], [24], the cellular consequence of *DJ-1β* deficiency in the oxidative stress response remains unclear. Furthermore, the link between DJ-1 and PD, especially in the context of oxidative stress, has not been thoroughly explored. In this study, we used *Drosophila* and mammalian cells to investigate the functional interaction between DJ-1 and its downstream target, Daxx/DLP. We also characterized the relationship between loss of *DJ-1* and PD-related phenotypes, such as DA neuronal degeneration and locomotive dysfunction, and examined the molecular mechanism of oxidative stress sensitivity in *Drosophila DJ-1* mutants.

## Results

### Oxidative stress-sensitive neuronal death in *DJ-1β* mutants

**Figure 1:**
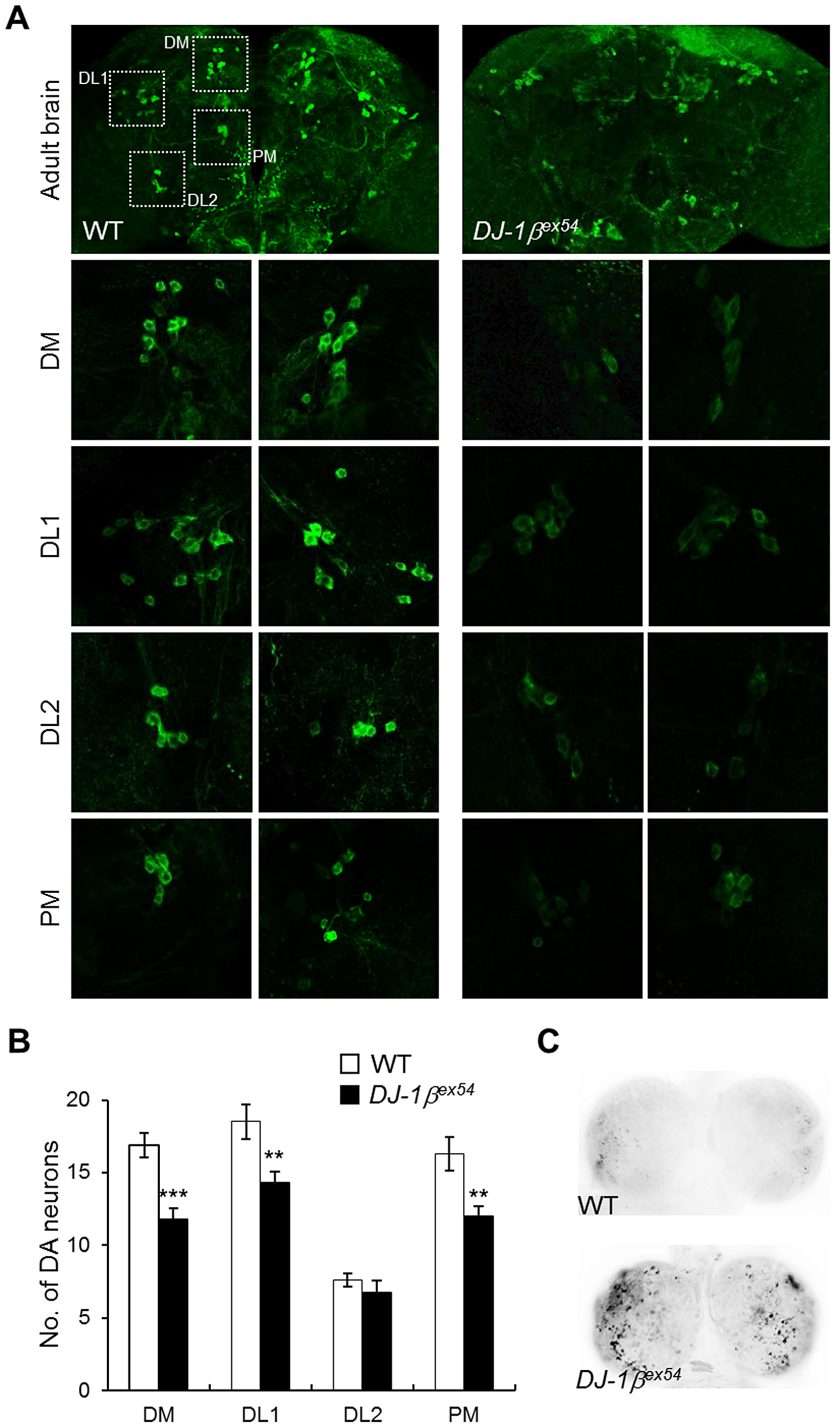
Decreased DA neurons and increased apoptosis in *DJ-1β* mutant under oxidative stress conditions. (A) DA neurons visualized by immunohistochemical analysis with anti-tyrosine hydroxylase antibody in the brains of wild-type (WT) and *DJ-1β* mutant (*DJ-1β^ex54^*) flies fed with 1% H_2_O_2_ for 3 days. Dotted boxed areas indicate DA neuron clusters. The lower pictures, including DM, DL1, DL2, and PM, are the magnified 4 dotted boxed areas of the upper pictures. Magnification of the upper pictures, 100×; Magnification of the lower pictures, 400×. (B) Graphs showing the number of DA neurons in each cluster of WT and *DJ-1β^ex54^* flies after feeding with H_2_O_2_ for 3 days (n = 10, Student's *t*-test: DM, *** p<0.001; DL1 and PM, ** p<0.01). The data are expressed as means ± s.e. values. (C) Acridine orange staining of 0.1% hydrogen peroxide-treated larval brains showed that increased oxidative stress-induced apoptosis in *DJ-1β^ex54^* compared to the WT controls. DM, dorsomedial clusters; DL, dorsolateral clusters; PM, posteriomedial clusters.

*Drosophila DJ-1β* mutants do not have gross morphological defects or loss of DA neurons when raised under standard laboratory conditions (Figure S1) [24]. However, expression level of tyrosine hydroxylase and number of DA neurons were significantly reduced in *DJ-1β* mutant flies after hydrogen peroxide treatment compared to those in wild-type animals (Figure 1A). The numbers of DA neurons in 3 major clusters of the posterior brain, dorsomedial clusters, dorsolateral clusters 1, and posteriomedial clusters, were significantly decreased by oxidative stress (Figure 1B). Next, we tested the effect of similar oxidative stress conditions on the neurons of larvae. As shown in Figure 1C, oxidative stress-induced cell death was dramatically increased in the brains of *DJ-1β* mutant larvae compared to that in wild-type controls. This suggests that *DJ-1β* mediates developmentally universal protection of DA neurons from oxidative stress-induced cell death.

### *DLP* is a downstream gene of *DJ-1β*

**Figure 2:**
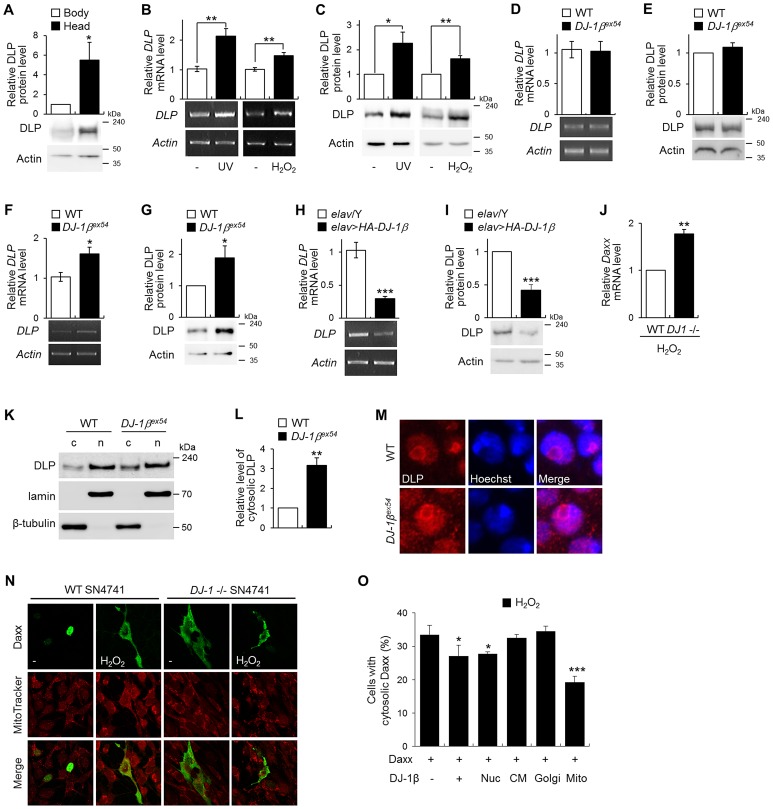
Gene expression of DLP by oxidative stress, UV, and *DJ-1β*. (A) DLP protein levels in the wild-type (WT) fly body and head (Student's *t*-test, n = 5, * p<0.05). (B–C) DLP mRNA (B) and protein (C) levels in WT embryos exposed to UV (50 mJ/cm^2^) and the heads of WT flies fed with 1% H_2_O_2_ (B, Student's *t*-test: UV, n = 5, ** p<0.01; H_2_O_2_, n = 5, ** p<0.01; C, Student's *t*-test: UV, n = 5, * p<0.05; H_2_O_2_, n = 4, ** p<0.01). (D–G) DLP mRNA (D, F) and protein (E, G) levels in the heads of WT and *DJ-1β^ex54^* flies grown with cornmeal-soybean standard fly food (D–E) and fed with 1% H_2_O_2_ for 3 days (F–G). (D, Student's *t*-test, n = 8; E, Student's *t*-test, n = 7; F, Student's *t*-test, n = 6, * p<0.05; G, Student's *t*-test, n = 7, * p<0.05). (H–I) DLP mRNA (H) and protein (I) levels in control (*elav*/Y) and pan-neuronally *DJ-1β*-overexpressing (*elav*>*HA-DJ-1β*) fly head (H, Student's *t*-test, n = 5, *** p<0.001; I, Student's *t*-test, n = 4, *** p<0.001). (J) *Daxx* mRNA level in the WT and *DJ-1* null SN4741 cells treated with 1 mM H_2_O_2_ (Student's *t*-test, n = 3, ** p<0.01). (K–L) Western blot (K) and statistical (L) analysis of cytosolic (c) and nuclear (n) fractions of WT and *DJ-1β^ex54^* fly head extracts showed that increased translocation of DLP to the cytosol in *DJ-1β^ex54^* compared to WT controls (Student's *t*-test, n = 4, ** p<0.01). Relative cytosolic DLP levels were calculated by dividing the normalized cytosolic DLP level by the normalized nuclear DLP level. Lamin and β-tubulin were used as loading controls for nuclear and cytosolic fractions, respectively. (M) Confocal images of DLP immunohistochemistry in the larval brains of WT and *DJ-1β^ex54^*. Hoechst-stained regions represent nuclei. Magnification, 8,000×. (N) Confocal images showing subcellular localization of Daxx in WT and *DJ-1* null SN4741 cells. MitoTracker-stained spots represent mitochondria. The cells were treated with 0.4 mM H_2_O_2_ for 1 h. (O) The ratio of the cells with cytosolic localized Daxx in *DJ-1* null SN4741 cells transfected with wild-type *DJ-1β* or nucleus (Nuc)-, cytoplasmic membrane (CM)-, Golgi (Golgi)-, or mitochondria (Mito)-targeted DJ-1β. The cells were treated with 0.4 mM H_2_O_2_ for 1 h. More than 70 cells per each samples were counted to calculate the ratio of the cells with cytosolic Daxx (Student's *t*-test, n = 4, * p<0.05, *** p<0.001). All data are expressed as means ± s.e. values. Actin was employed as an internal control of total extract.

To characterize the protective mechanism of DJ-1β, we used microarrays to compare the gene expression profiles of *DJ-1β* mutants and wild-type controls under oxidative stress conditions. We identified 143 upregulated and 134 downregulated genes (>1.5-fold changes in expression *DJ-1β* mutants) in the mRNA extracted from fly heads (Table S1). The role of *DLP* in the DJ-1-dependent oxidative stress response was first examined among the upregulated genes because *Daxx*, the mammalian homologue of *DLP*, has been implicated in oxidative stress-induced apoptosis [40] and identified as a potential component of PD pathogenesis [33], [44]. DLP is a 183.9-kDa protein with approximately 46% similarity to human Daxx in the Daxx-homology region; it is the only Daxx homologue in the *Drosophila* genome [45]. Interestingly, DLP protein levels were significantly higher in the fly head than in the body (Figure 2A and Figure S2), suggesting that it performs an important function in the brain. Furthermore, DLP mRNA and protein levels were increased by both UV irradiation and oxidative stress (Figure 2B and 2C), implying that DLP is involved in these stress responses, similar to its mammalian counterpart.

To confirm our microarray data, we evaluated *DLP* expression in the heads of wild-type and *DJ-1β* mutant flies following treatment with H_2_O_2_ using real-time quantitative PCR and western blot analysis. The levels of DLP mRNA and protein did not differ between the *DJ-1β* mutants and wild-type controls under standard laboratory conditions (Figure 2D and 2E). However, as anticipated, the levels of DLP mRNA and protein were significantly elevated in *DJ-1β* mutants in comparison to wild-type controls under oxidative stress (Figure 2F and 2G), suggesting that the increasing rate of *DLP* expression by oxidative stress in *DJ-1β* mutants is higher than that in wild type. When *DJ-1β* was overexpressed with a pan-neuronal *elav*-*GAL4* driver, the levels of DLP transcript and protein were significantly reduced (Figure 2H and 2I). Interestingly, mammalian *Daxx* gene expression was also higher in *DJ-1* null cells than in wild-type controls under oxidative stress condition (Figure 2J). These results indicate that DJ-1 functions as a negative regulator of *Daxx*/*DLP* gene expression under oxidative stress conditions.

### Altered subcellular localization of DLP in *DJ-1β* mutants

As mammalian DJ-1 inhibits translocation of the nuclear Daxx to the cytosol [33], [46], we examined whether *Drosophila* DJ-1β also regulates subcellular localization of DLP. First, we fractionated DLP protein from the cytosol and nucleus of wild-type and *DJ-1β* mutant fly heads (Figure 2K). The proportion of cytosolic DLP relative to nucleic DLP was increased more than 3-fold in *DJ-1β* fly heads (Figure 2K and 2L). Consistently, immunohistochemical analysis with anti-DLP antibody showed that the cytosolic DLP level was significantly increased in the brain and eye imaginal disc of *DJ-1β* mutant flies compared to that in wild-type flies (Figure 2M and Figure S3, respectively), which was highly similar in *DJ-1* null mouse DA neuroblastoma cells (Figure 2N).

Since we previously showed that DJ-1 is partially localized in mitochondria [24], we examined whether mitochondrial translocation of DJ-1 is important for the cytosolic localization of Daxx by comparing the effect of several forms of DJ-1 that are targeted to various subcellular regions including mitochondria, Golgi, nucleus, and cytoplasmic membrane. Interestingly, the mitochondrial DJ-1β efficiently inhibited translocation of Daxx from the nucleus to the cytosol under oxidative stress conditions, like wild-type or nucleus targeted DJ-1β (Figure 2O and Figure S4). These results suggest that DJ-1 regulates the translocation of Daxx/DLP as well as their gene expression in response to oxidative stress.

### DLP mediates oxidative stress- and UV-induced apoptosis

**Figure 3:**
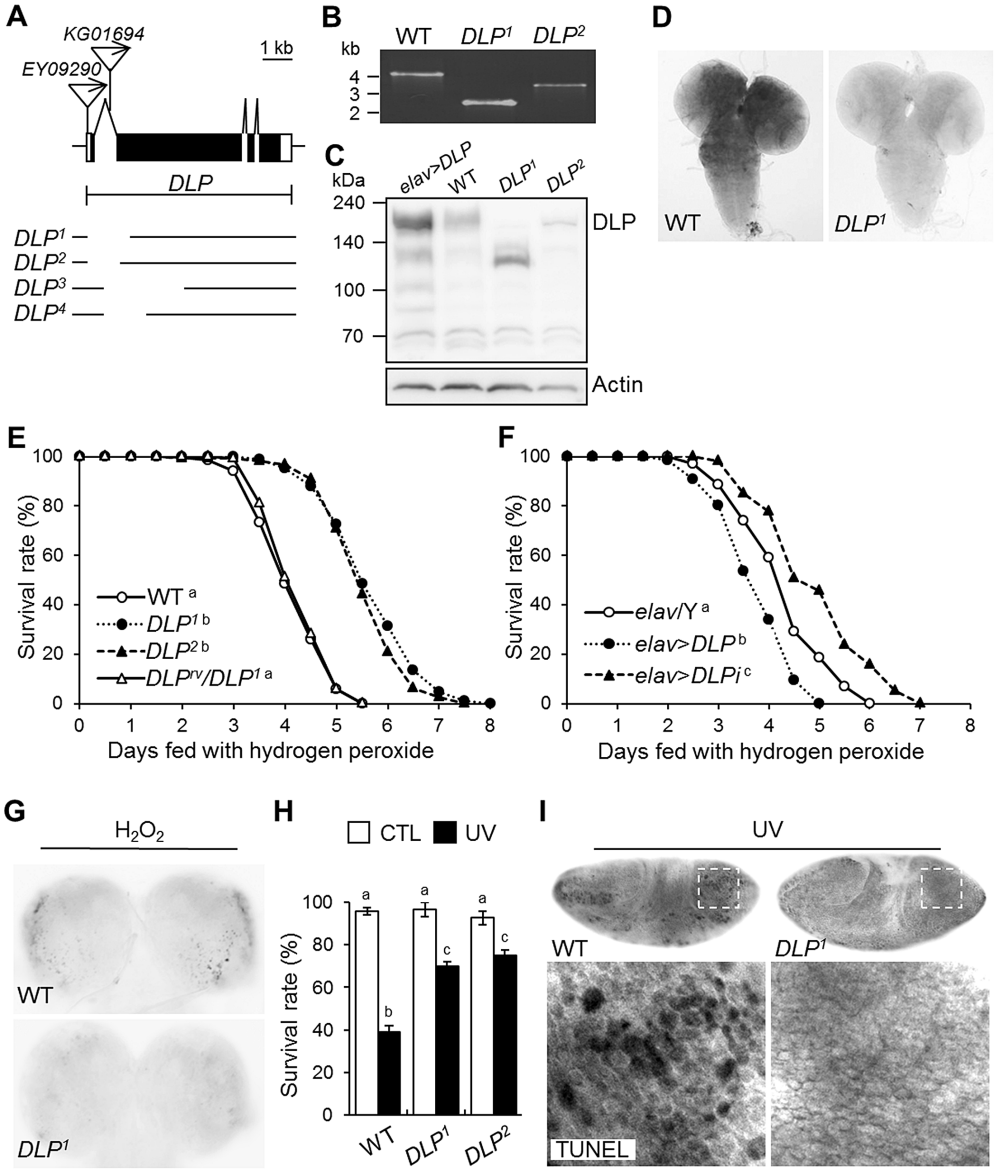
Generation and characterization of *DLP* mutants. (A) Genomic structure of the *DLP* gene. Exons of the *DLP* gene are shown in black (coding region) and white (non-coding region) boxes. The inverted triangles indicate the P-elements, *EY09290* and *KG01694*. The deletion sites of *DLP^1^*, *DLP^2^*, *DLP^3^*, and *DLP^4^* are illustrated under the genomic structures. (B) Determination of the deleted size in *DLP* mutants by genomic DNA PCR. (C) Western blotting of DLP in wild type (WT), *DLP* loss-of-function (*DLP^1^* and *DLP^2^*) and gain-of-function (*elav*>*DLP*) mutants. Intact DLP protein is not detected in the *DLP* mutants. (D) Comparison of *DLP* gene expression in the third-instar larval brains of WT and a *DLP* mutant via RNA *in situ* hybridization. (E–F) Survival rates of *DLP* loss-of-function (*DLP^1^*, *DLP^2^*, and *elav*>*DLPi*) and gain-of-function (*elav*>*DLP*) mutants under oxidative stress conditions. (E) WT and *DLP^rv^/DLP^1^* were used as controls (log-rank test: WT, n = 300; *DLP^1^*, n = 250; *DLP^2^*, n = 250; *DLP^rv^/DLP^1^*, n = 300, p<0.01, groups with the same letter do not differ significantly). (F) *elav*/Y was used as a control (log-rank test: *elav*/Y, n = 350; *elav*>*DLP*, n = 300; *elav*>*DLPi*, n = 300, p<0.01, groups with the different letter differ significantly). The genotypes of the samples were *elav*/Y (*elav-GAL4*/Y), *elav*>*DLP* (*elav-GAL4*/Y; *EY09290*/+), and *elav*>*DLPi* (*elav-GAL4*/Y; *UAS*-*DLP-RNAi*/+). (G) Acridine orange staining of larval brains of *DLP^1^* and WT treated with 0.1% H_2_O_2_ for 24 h. (H) Survival rates of WT and *DLP* mutant (*DLP^1^* and *DLP^2^*) pupae after exposure to UV irradiation (10 mJ/cm^2^; black bars) as described in the Materials and Methods (Kruskal-Wallis test: CTL, n≥6, p<0.1; UV, n = 6, p<0.01, groups with the same letter do not differ significantly). CTL, UV-untreated control pupae; UV, UV-treated pupae. All data are expressed as means ± s.e. values. (I) TUNEL-stained images of UV-exposed 0–3 h embryos of WT and *DLP^1^*. The lower panels are higher-magnification images of the boxes indicated with dotted lines in the upper panels. CTL, control; rv, revertant; TUNEL, terminal deoxynucleotidyl transferase-mediated dUTP-biotin nick end-labeling.

To evaluate the role of DLP in the hypersensitivity of *DJ-1β* mutants to oxidative stress and UV irradiation, we generated and characterized *DLP* mutants. The mutant *EY09290*, which harbors a P-element inserted in the 5′ region of the *DLP* gene, was acquired from the Bloomington *Drosophila* Stock Center, and deletion mutants were generated via P-element mobilization (Figure 3A). Two deletion alleles were generated and designated *DLP^1^* and *DLP^2^*. The genomic deletions were confirmed by PCR (Figure 3B) and DNA sequencing. These alleles have deletions of 1,311 and 1,076 bp, respectively, which removes the first 2 exons, including the translational start site of *DLP* (Figure 3A). Western blot analysis and RNA *in situ* hybridization results confirmed that DLP protein and mRNA levels were markedly reduced in *DLP^1^* and *DLP^2^* compared to the levels in wild-type controls (Figure 3C and 3D). In order to exclude the genetic background effect, we generated two other *DLP* deletion mutants, *DLP^3^* and *DLP^4^*, using another P-element line, *KG01694* (Figure 3A), and we obtained another *DLP* mutant, *DLP^U42^*, with a different genetic lineage [45]. Therefore, we used five mutant alleles in three different genetic backgrounds to characterize the *DLP* mutants. Furthermore, *Upstream Activation Sequence* (*UAS*)-*DLP*-*RNAi* was used for *DLP* knockdown to confirm the *DLP* mutant phenotypes.

Because Daxx has been implicated in oxidative stress and UV responses, the role of DLP in oxidative stress-induced lethality was assessed using these *DLP* mutants. As expected, *DLP* mutants proved more resistant to H_2_O_2_ treatment than wild-type or *trans*-heterozygotes between *DLP* revertant (*DLP^rv^*) and *DLP^1^* (Figure 3E and Figure S5). Moreover, *DLP* knockdown in all neurons using *UAS*-*DLP*-*RNAi* conferred increased resistance to oxidative stress, whereas *DLP* overexpression in the same neurons increased their sensitivity to oxidative stress (Figure 3F). Oxidative stress-induced cell death was also attenuated in the *DLP* mutant brain (Figure 3G). UV irradiation-induced pupal lethality and apoptosis were also significantly reduced in *DLP* mutants (Figure 3H and 3I), indicating that DLP is involved in UV-induced stress responses. These results indicate that the neuronal function of DLP is important to the oxidative and UV stress responses at both cellular and organism level.

### *DLP* functions as a pro-apoptotic gene by activating the JNK/dFOXO signaling pathway

**Figure 4:**
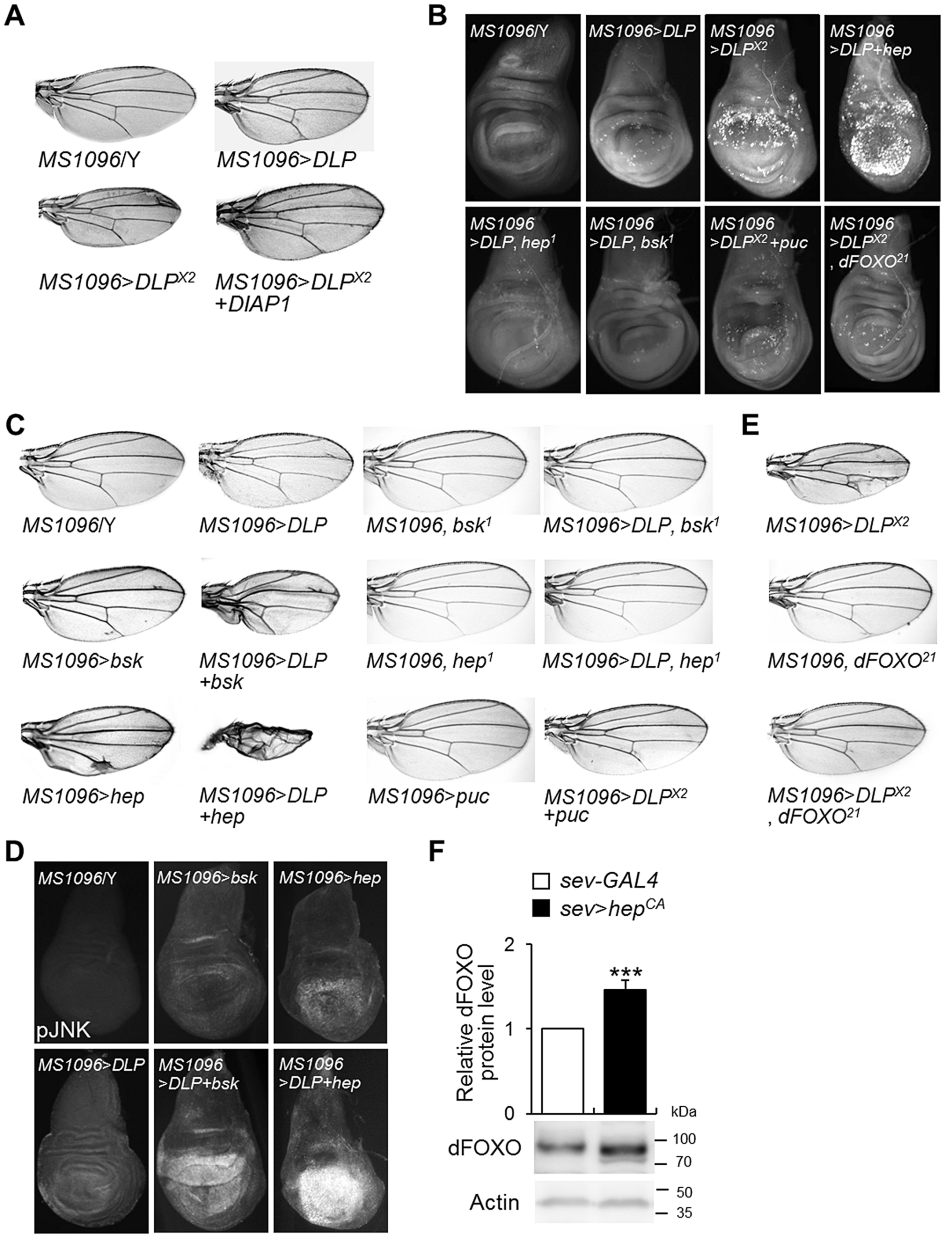
DLP activates apoptosis and the JNK/dFOXO signaling pathway. (A) Comparison of tissue sizes of control (*MS1096*/Y), *DLP*-overexpressing (*MS1096*>*DLP* and *MS1096*>*DLP* ^×*2*^), and *DLP*- and *DIAP1*-coexpressing (*MS1096*>*DLP* ^×*2*^+*DIAP1*) fly wings. Two copies of the *DLP* gene were overexpressed in *MS1096*>*DLP* ^×*2*^. (B) Acridine orange-stained images of control (*MS1096*/Y), *DLP*-overexpressing (*MS1096*>*DLP* and *MS1096*>*DLP* ^×*2*^), *DLP*- and *hep*-coexpressing (*MS1096*>*DLP*+*hep*), *DLP*-overexpressing and *hep* deficient (*MS1096*>*DLP*, *hep^1^*), *DLP*-overexpressing and *bsk* deficient (*MS1096*>*DLP*, *bsk^1^*), *DLP*- and *puc*-coexpressing (*MS1096*>*DLP* ^×*2*^+*puc*), and *DLP*-overexpressing and *dFOXO* deficient (*MS1096*>*DLP* ^×*2*^, *dFOXO^21^*) wing imaginal discs. (C) Genetic interactions of *DLP* with *bsk*, *hep*, and *puc* in the developing wing. The reduced wing phenotype induced by *DLP* overexpression (*MS1096*>*DLP*) was strongly exacerbated by *bsk* (*MS1096*>*DLP*+*bsk*) or *hep* (*MS1096*>*DLP*+*hep*) overexpression, and suppressed by *bsk* (*MS1096*>*DLP*, *bsk^1^*) or *hep* (*MS1096*>*DLP*, *hep^1^*) deficiency or co-expression of *puc* (*MS1096*>*DLP* ^×*2*^+*puc*). (D) Comparison of JNK activity in the *DLP*- and *bsk*-coexpressing or *DLP*- and *hep*-coexpressing wing imaginal discs (*MS1096*>*DLP*+*bsk* or *MS1096*>*DLP*+*hep*) with *DLP*-, *bsk*- or *hep*-overexpressing wings (*MS1096*>*bsk* or *MS1096*>*hep*) by anti-phospho-JNK antibody staining. (E) Genetic interactions of *DLP* with *dFOXO* in the developing wing. The wing phenotype of *DLP* overexpression (*MS1096*>*DLP* ^×*2*^) was strongly suppressed by *dFOXO* deficiency (*MS1096*>*DLP* ^×*2*^, *dFOXO^21^*). *MS1096* with *dFOXO* deficiency (*MS1096*, *dFOXO^21^*) was used as controls. (F) DLP protein levels in the control (*sev-GAL4*) and constitutive active *hep*-overexpressing (*sev*>*hep^CA^*) fly heads (Student's *t*-test, n = 9, *** p<0.001). *sev*, *sevenless-GAL4*. The data are expressed as means ± s.e. values. The genotypes of the samples were *MS1096*/Y (*MS1096-GAL4*/Y), *MS1096*>*bsk* (*MS1096-GAL4*/Y; *UAS-bsk*/+), *MS1096*>*hep* (*MS1096-GAL4*/Y; *UAS-hep*/+), *MS1096*, *bsk^1^* (*MS1096-GAL4*/Y; *bsk^1^*/+), *MS1096*, *hep^1^* (*MS1096-GAL4*/*hep^1^*), *MS1096*>*DLP* (*MS1096-GAL4*/Y; *EY09290*/+), *MS1096*>*DLP*+*bsk* (*MS1096-GAL4*/Y; *EY09290*/*UAS-bsk*), *MS1096*>*DLP*+*hep* (*MS1096-GAL4*/Y; *EY09290*/*UAS-hep*), *MS1096*>*DLP*, *bsk^1^* (*MS1096-GAL4*/Y; *EY09290*/*bsk^1^*), *MS1096*>*DLP*, *hep^1^* (*MS1096-GAL4*/*hep^1^*; *EY09290*/+), *MS1096*>*DLP* ^×*2*^ (*MS1096-GAL4*/Y; *EY09290*/*EY09290*), *MS1096*>*puc* (*MS1096-GAL4*/Y; *UAS-puc*/+), *MS1096*>*DLP* ^×*2*^+*puc* (*MS1096-GAL4*/Y; *EY09290*/*EY09290*; *UAS-puc*/+), *MS1096*, *dFOXO^21^* (*MS1096-GAL4*/Y;; *dFOXO^21^*/+), *MS1096*>*DLP* ^×*2*^, *dFOXO^21^* (*MS1096-GAL4*/Y; *EY09290*/*EY09290*; *dFOXO^21^*/+), MS1096>*DLP* ^×*2*^+*DIAP1* (*MS1096-GAL4*/Y; *EY09290*/*EY09290*; *UAS-DIAP1*/+), *sev-GAL4* (*sev-GAL4*/+), and *sev*>*hep^CA^* (*sev-GAL4*/+; *UAS-hep^CA^*/+). *bsk*, *basket*; *DIAP1*, *Drosophila inhibitor of apoptosis protein 1*; *hep*, *hemipterous*; pJNK, phospho-JNK; *puc*, *puckered*.

*Daxx* was originally identified as a pro-apoptotic gene that induced cell death [35]. However, the role of Daxx and DLP in apoptosis is somewhat controversial [38], [45]. In order to confirm the function of DLP in non-stress-induced apoptosis, we utilized the *UAS-GAL4* system to evaluate the effects of *DLP* overexpression on developing tissues. As *EY09290* harbors a P-element with *UAS* in the 5′ region of *DLP* and the direction of the P-element is oriented to induce *DLP* gene expression (Figure 3A), the mutant was employed to study *DLP* overexpression. The induction of *DLP* expression was confirmed by RNA *in situ* hybridization in the wing imaginal discs of the *EY09290* line harboring *MS1096*-*GAL4* driver (Figure S6A). Interestingly, *DLP* overexpression in the developing wing under the control of *MS1096-GAL4* reduced organ size in a dose-dependent manner (Figure 4A). When *DLP* was overexpressed in neurons, reduced survival and defects in locomotive behavior were observed (Figure S6B and S6C), suggesting that increased *DLP* expression has an adverse effect on neuronal development and function in *Drosophila*. Indeed, *DLP* overexpression strongly induced cell death in the imaginal disc (Figure 4B; compared with *MS1096*/Y and *MS1096*>*DLP* ^×*2*^) without affecting the cell cycle or differentiation (Figure S6D and S6E). Furthermore, the *DLP*-induced reduction in wing size was suppressed almost completely by co-expression of *Drosophila inhibitor of apoptosis protein 1* (*DIAP1*; Figure 4A), a caspase inhibitor. These results demonstrate that DLP activity induces apoptosis through caspase activation and reduces overall survival, as demonstrated by the reduced survival of *DLP*-overexpressing flies (Figure S6B).

In mammals, Daxx induces apoptosis by activating the JNK signaling pathway [35], [39]. We attempted to determine whether the JNK signaling pathway is activated by DLP in *Drosophila*. We initially examined the genetic interaction of *DLP* with *basket* (*bsk*), a *Drosophila JNK*, and *hemipterous* (*hep*), a *Drosophila JNK kinase* (*JNKK*). Although overexpression of *DLP*, *bsk*, or *hep* in the wing produced only a slight reduction in wing size, overexpression of either *bsk* or *hep* in conjunction with *DLP* resulted in severely rumpled and shrunken wings (Figure 4C). Consistently, co-expression of *hep* with *DLP* strongly induced cell death in the wing imaginal disc (Figure 4B). On the other hand, *bsk* or *hep* loss-of-function mutation or co-expression of *puckered* (*puc*), a negative regulator of JNK (a JNK phosphatase), suppressed the cell death and wing deformities induced by *DLP* overexpression (Figure 4B and 4C). Furthermore, co-expression of *bsk* or *hep* with *DLP* resulted in a strong increase of JNK phosphorylation (Figure 4D). These results demonstrate that DLP activates the JNK signaling pathway, similar to mammalian Daxx.

As FOXO is a target of JNK in mammalian systems [47] and acts downstream of JNK signaling in the control of apoptosis [48], we investigated the role of FOXO in *DLP*-induced apoptosis. We examined the effect of *FOXO* deficiency on the *DLP*-induced gain-of-function phenotype and apoptosis. *FOXO* deficiency (*dFOXO^21^*) suppressed the *DLP*-induced wing (Figure 4E) and cell death (Figure 4B) phenotypes, suggesting that the JNK/dFOXO pathway is downstream of DLP.

It has been shown that JNK activates FOXO4 activity through phosphorylation at Thr447 and Thr451 [47]. Therefore, we assessed whether dFOXO is also phosphorylated by JNK. Amino acid sequence analysis between mammalian FOXOs and dFOXO did not reveal the conserved Thr447 and Thr451 phosphorylation sites in dFOXO. Furthermore, we could not see any evidence to support direct phosphorylation of dFOXO by JNK in *in vitro* phosphorylation experiments (data not shown). However, interestingly, expression of constitutively active JNKK (*hep^CA^*) significantly increased the protein level of dFOXO (Figure 4F), implicating that JNK regulates dFOXO activity by increasing its protein level or stability in *Drosophila*.

### DLP contributes to the oxidative stress-related phenotypes of *DJ-1β* mutants

**Figure 5:**
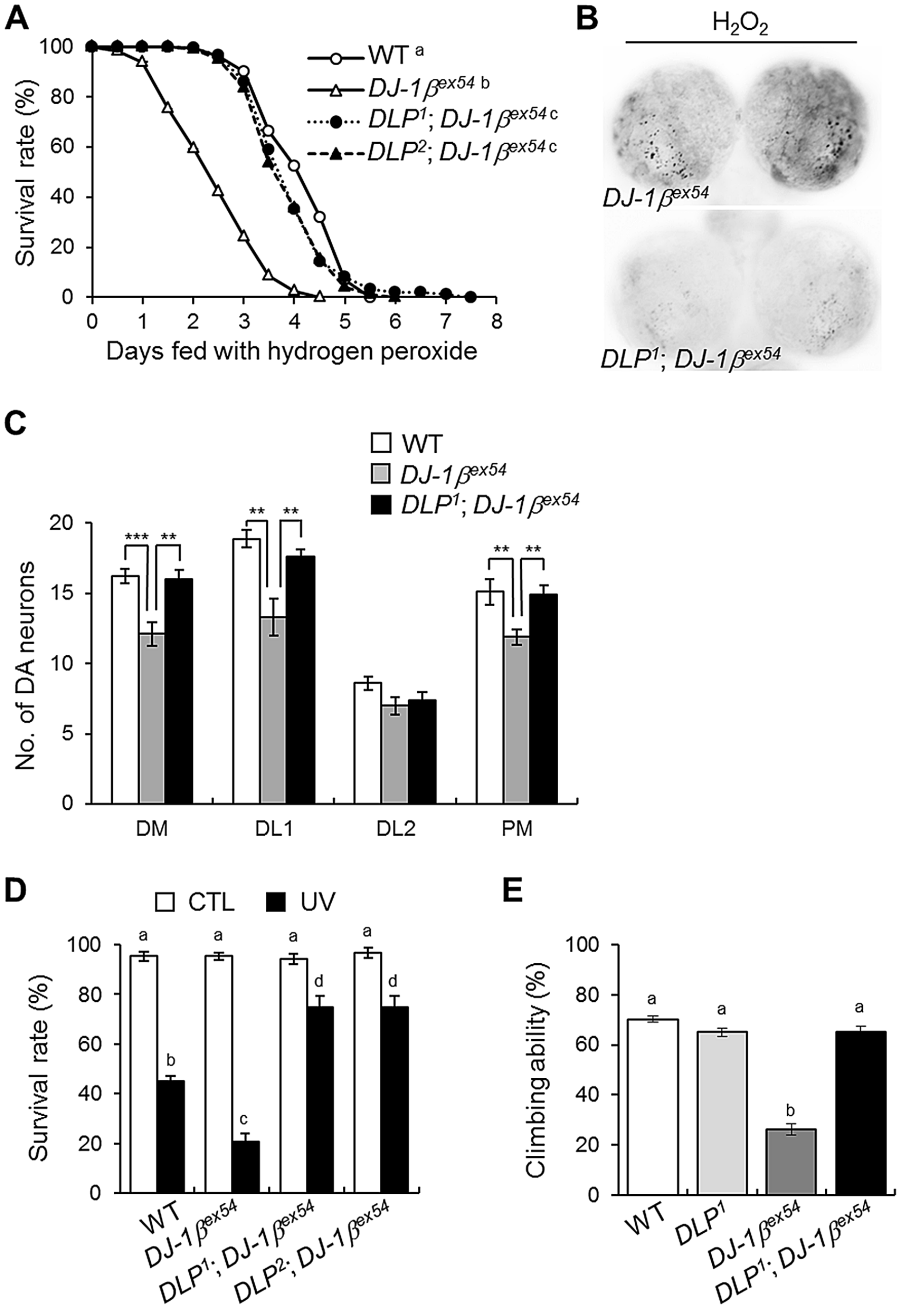
*DLP* deficiency reduces acute sensitivity to oxidative stress and UV, and improves locomotive dysfunction in *DJ-1β* mutant. (A) Comparison of the survival rates of *DLP* and *DJ-1β* double mutants (*DLP^1^*; *DJ-1β^ex54^* and *DLP^2^*; *DJ-1β^ex54^*) with wild-type (WT) and *DJ-1β^ex54^* flies under oxidative stress conditions (log-rank test: WT, n = 250; *DJ-1β^ex54^*, n = 250; *DLP^1^*; *DJ-1β^ex54^*, n = 250; *DLP^2^*; *DJ-1β^ex54^*, n = 250, p<0.01, groups with the same letter do not differ significantly). (B) Reduced oxidative stress-induced apoptosis was noted in the larval brain of the *DLP* and *DJ-1β* double mutant (*DLP^1^*; *DJ-1β^ex54^*) compared to *DJ-1β^ex54^*. The larval brains were treated with 0.1% H_2_O_2_ for 24 h and cell death was detected via acridine orange staining. (C) Sensitized DA neuronal death of *DJ-1β^ex54^* under oxidative stress conditions was rescued by *DLP* deficiency. The flies were fed with 1% H_2_O_2_ for 3 days. (n = 10, Student's *t*-test: DM, ** p<0.01, *** p<0.001; DL1, ** p<0.01; PM, ** p<0.01). (D) Survival rates of WT, *DJ-1β^ex54^*, and double mutant of *DLP* and *DJ-1β* (*DLP^1^*; *DJ-1β^ex54^* and *DLP^2^*; *DJ-1β^ex54^*) pupae after exposure to UV irradiation (10 mJ/cm^2^; black bars) as described in the Materials and Methods (Kruskal-Wallis test: CTL, n≥6, p>0.1; UV, n≥5, p<0.01, groups with the same letter do not differ significantly). CTL, UV-untreated control pupae; UV, UV-treated pupae. (E) Comparison of climbing abilities of WT, *DLP^1^*, *DJ-1β^ex54^*, and double mutants of *DLP* and *DJ-1β* (*DLP^1^*; *DJ-1β^ex54^*). The climbing abilities of 5-day-old flies for each group were tested as described in the Materials and Methods (ANOVA and Tukey's HSD analysis: n≥12, p<0.01, groups with the same letter do not differ significantly). All data are expressed as means ± s.e. values.

Because *DLP* expression is regulated by DJ-1β under oxidative stress conditions (Figure 2) and DLP is important for oxidative stress-induced apoptosis and lethality (Figure 3 and Figure S5), we assessed the role of DLP in the oxidative stress-related phenotypes of *DJ-1β* mutants, specifically their acute sensitivity to oxidative stress and locomotive dysfunction. To accomplish this, we generated *DLP* and *DJ-1β* double mutants and asked whether *DLP* deficiency could rescue various *DJ-1β* mutant phenotypes. We first evaluated the H_2_O_2_ sensitivity of these lines. As shown in Figure 5A, *DLP* and *DJ-1β* double mutants displayed survival rates similar to those of wild-type controls, whereas *DJ-1β* mutants were acutely sensitive to H_2_O_2_. However, the survival rates of *DLP* and *DJ-1β* double mutants was still lower than those of *DLP* mutants (compare Figure 5A with Figure 3E), which suggests that the H_2_O_2_ sensitivity of *DJ-1β* is not fully dependent on DLP. Supporting this, gene expression of 18 oxidative stress-related genes (11 up-regulated and 7 down-regulated) was altered in *DJ-1β* mutants versus wild-type controls (Table S1). Although DLP is not expected to mediate the whole effect of the oxidative stress responses in *DJ-1β* mutants, *DLP* deficiency strongly suppressed the oxidative stress-induced cell death observed in *DJ-1β* mutant brains (Figure 5B). Consistently, oxidative stress-induced DA neuronal death in *DJ-1β* mutants was almost completely inhibited by *DLP* deficiency (Figure 5C and Figure S7). These findings indicate that DLP is a key mediator in the oxidative stress-induced neuronal cell death of *DJ-1β* mutants. Furthermore, *DLP* deficiency rescued the UV sensitivity (Figure 5D) and locomotive dysfunction (Figure 5E) of *DJ-1β* mutants. These results strongly suggest that DLP mediates the H_2_O_2_-induced oxidative stress responses, UV sensitivity, and locomotive dysfunction of *DJ-1β* mutants.

### DJ-1β negatively regulates *DLP* expression through dFOXO

**Figure 6:**
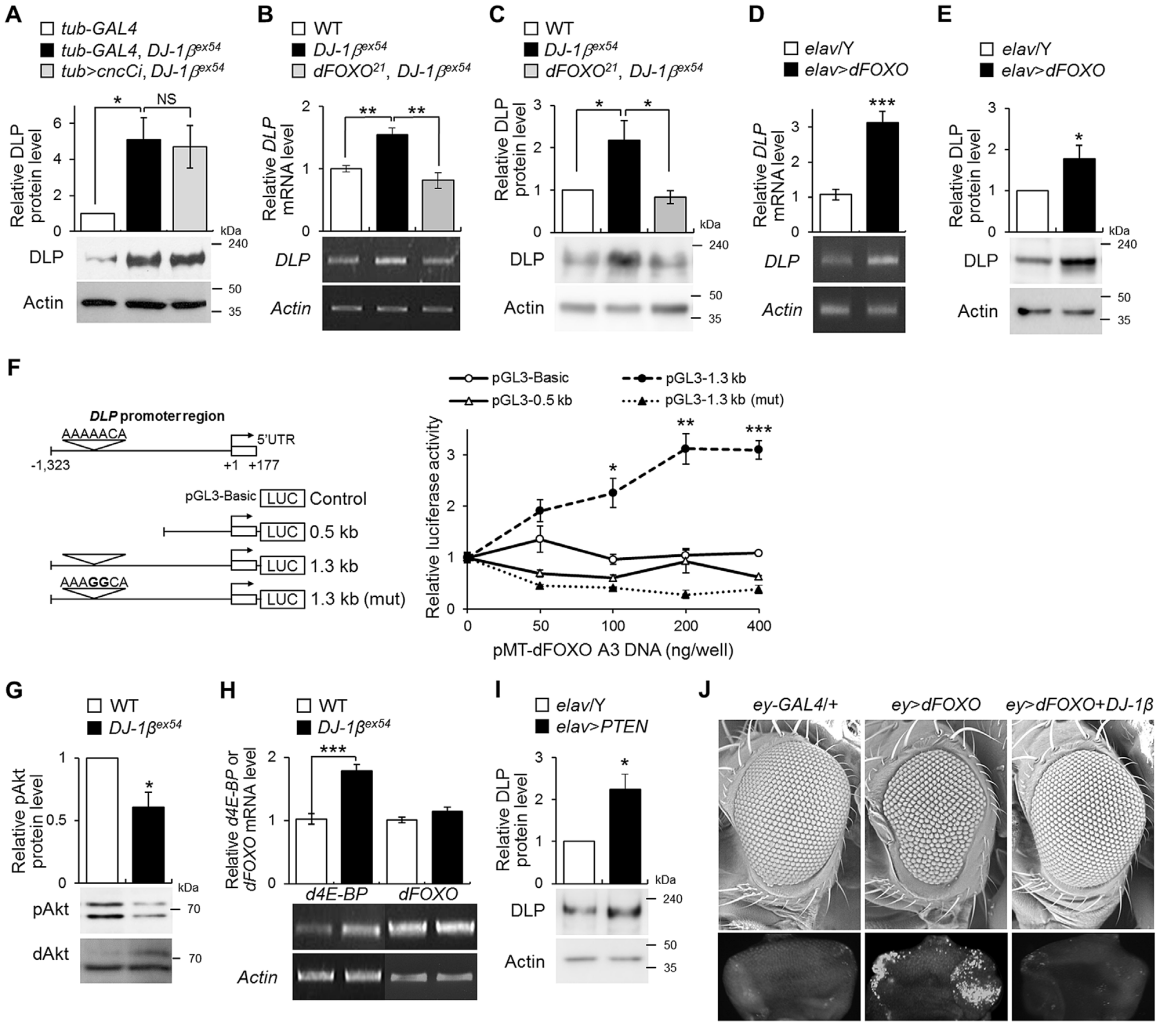
The role of *dFOXO* in the regulation of *DLP* by *DJ-1β*. (A) DLP protein levels in the heads of control (*tub*-*GAL4*), *DJ-1β* mutant (*tub*-*GAL4*, *DJ-1β^ex54^*) and the double mutant of *cncC* and *DJ-1β* (*tub*>*cncCi*, *DJ-1β^ex54^*) flies fed with 1% H_2_O_2_ (Student's *t*-test, n = 4, * p<0.05). NS, not significant. (B–C) DLP mRNA (B) and protein (C) levels in the head of WT, *DJ-1β^ex54^*, and *dFOXO* and *DJ-1β* double mutant (*dFOXO^21^*, *DJ-1β^ex54^*) flies fed with 1% H_2_O_2_ (B, Student's *t*-test, n = 5, ** p<0.01; C, Student's *t*-test, n = 4, * p<0.05). (D–E) DLP mRNA (D) and protein (E) levels in control (*elav*/Y) and pan-neuronally *dFOXO*-overexpressing (*elav*>*dFOXO*) fly heads (D, Student's *t*-test, n = 6, *** p<0.001; E, Student's *t*-test, n = 5, * p<0.05). (F) Luciferase assays showed activation of *DLP* promoters in S2 cells after cotransfection with dFOXO-A3. (Open circle) Empty vector. (Open triangle) 0.5-kb fragment of *DLP* promoter. (Filled circle) 1.3-kb fragment of *DLP* promoter. (Filled triangle) 1.3-kb fragment of *DLP* promoter with mutation in the putative FRE site: pGL3–1.3 kb (mut). Bold characters in the putative FRE site represent the mutated nucleotides. (Student's *t*-test, n = 3, * p<0.05; ** p<0.01; *** p<0.001). (G) The levels of phospho-Akt in the head of WT and *DJ-1β^ex54^* flies fed with 1% H_2_O_2_ (Student's *t*-test, n = 3, * p<0.05). dAkt was used as an internal control. (H) *d4E-BP*, a target of dFOXO, and *dFOXO* mRNA levels in the head of WT and *DJ-1β^ex54^* flies fed with 1% H_2_O_2_ (Student's *t*-test: *d4E-BP*, n = 7, *** p<0.001; *dFOXO*, n = 7). (I) DLP protein levels in the control (*elav*/Y) and pan-neuronally *PTEN*-overexpressing (*elav*>*PTEN*) fly heads (Student's *t*-test, n = 4, * p<0.05). (J) Genetic interactions of *dFOXO* with *DJ-1β* in the developing eye. The upper pictures are scanning electron micrographs of the fly eyes. The lower pictures are acridine orange-stained images of the eye imaginal discs. The genotypes of the samples were *tub*-*GAL4* (*tub*-*GAL4*/+), *tub*-*GAL4*, *DJ-1β^ex54^* (*tub*-*GAL4*, *DJ-1β^ex54^*/*DJ-1β^ex54^*), *tub*>*cncCi*, *DJ-1β^ex54^* (*UAS*-*cncC*-*RNAi*/+; *tub*-*GAL4*, *DJ-1β^ex54^*/*DJ-1β^ex54^*), *dFOXO^21^*, *DJ-1β^ex54^* (*dFOXO^21^*, *DJ-1β^ex54^*/*DJ-1β^ex54^*), *elav*/Y (*elav-GAL4*/Y), *elav*>*dFOXO* (*elav-GAL4*/Y; *UAS-dFOXO*/+), *elav*>*PTEN* (*elav-GAL4*/Y; *UAS-PTEN*/+), *ey-GAL4* (*ey-GAL4*/+), *ey*>*dFOXO* (*UAS-dFOXO*/+; *ey-GAL4*/+), and *ey*>*dFOXO*+*DJ-1β* (*UAS-dFOXO*/*UAS-HA-DJ-1β*; *ey-GAL4*/+). pAkt, phospho-Akt; *ey*, *eyeless*. All data are expressed as means ± s.e. values. Actin was used as an internal control.

Next, we investigated the mechanism by which DJ-1β regulates *DLP* expression. Previous studies with mammalian DJ-1 suggest that DJ-1 affects oxidative stress-related gene expression by stabilizing Nrf2 [29] or by suppressing p53 transcriptional activity [34]. Alternatively, DJ-1 regulates phosphatidylinositol 3-kinase (PI3K)/Akt signaling [19], [49], [50], which inhibits FOXO. Therefore, we tested whether *cap'n'collar C* (*cncC*, *Drosophila* Nrf2), *p53*, or dFOXO is involved in the regulation of *DLP* gene expression by DJ-1β under oxidative stress conditions. As demonstrated in Figure 6A and Figure S8, neither *cncC* nor *p53* affected the DLP levels in wild-type or *DJ-1β* mutant flies. However, the increased DLP mRNA (Figure 6B) and protein (Figure 6C) levels in *DJ-1β* mutants were restored to normal levels by *dFOXO* deficiency, suggesting dFOXO is required for elevation of *DLP* expression in *DJ-1β* mutants. Thus, when *dFOXO* was overexpressed in neurons, DLP expression was elevated more than 2-fold for both mRNA (Figure 6D) and protein (Figure 6E). Since a putative FOXO recognition element (FRE, AAAAACA) is located at 1,041 bp upstream of the transcription start site of the *DLP* gene (Figure 6F) [51], to examine the positive effect of dFOXO on the *DLP* gene expression at the transcriptional level, we cloned two different sizes of the *DLP* promoter region into a firefly reporter plasmid; 0.5 kb and 1.3 kb, respectively. Upon transient co-transfection with a construct expressing the constitutively active form of dFOXO (pMT-dFOXO A3), the construct containing the 1.3-kb fragment of the promoter region, but not the 0.5-kb fragment, exhibited the dFOXO-dependant promoter activity in a dose dependent manner (Figure 6F). To confirm whether this putative FRE site is critical, a mutation that disrupts the dFOXO binding was introduced into the 1.3-kb promoter construct by site-directed mutagenesis. Upon transfection, the construct containing the mutant 1.3-kb promoter region no longer showed dFOXO-dependent promoter activity (Figure 6F), indicating that dFOXO regulates *DLP* expression through this FRE site.

Additionally, we revealed that phospho-Akt (an activated form of *Drosophila* Akt (dAkt), a negative regulator of dFOXO) was significantly reduced in *DJ-1β* mutants (Figure 6G). We also found that gene expression of *Drosophila 4E-BP*, a target of dFOXO, increased in *DJ-1β* mutants, although *dFOXO* gene expression remained unaltered (Figure 6H). Moreover, overexpression of *PTEN*, a negative regulator of the Akt signaling pathway and, therefore, an activator of dFOXO, elevated the DLP level (Figure 6I). These results consistently indicate that DJ-1β regulates *DLP* gene expression through the PI3K-Akt-dFOXO pathway.

Finally, we investigated whether DJ-1β could suppress dFOXO-induced apoptosis. As shown in Figure 6J, *DJ-1β* overexpression strongly suppressed dFOXO-induced eye degeneration and apoptosis (compare *ey*>*dFOXO* with *ey*>*dFOXO*+*DJ-1β*).

## Discussion

Recently, several *Drosophila* PD models, each of which represents a different PD-associated gene mutant, have been developed and characterized [10]–[13], [16], [24], [49], [52]–[54]. Although each exhibits a distinct phenotype, a common feature of all these models is sensitization to oxidative stress. This, along with pathology data from PD patients [55]–[57], strongly indicates a significant role for oxidative stress in the development and progression of PD. Since the *DJ-1* mutations have been linked to familial PD and hypersensitivity to toxins that induce oxidative stress [23], we examined a signaling pathway that controls oxidative stress responses by DJ-1 in *Drosophila*. We demonstrated that DJ-1β inhibits oxidative stress-induced neuronal apoptosis by regulating DLP gene expression and protein subcellular localization, suggesting a causal relationship between *DJ-1β* mutation and oxidative stress-induced DA neuronal loss in PD.

Our genetic and cellular analyses indicate *DLP* functions as a pro-apoptotic gene and as a JNK activator in *Drosophila*, like its mammalian homologue *Daxx*. Previous studies have shown that Daxx is upregulated in response to oxidative stress and UV irradiation; it also mediates apoptosis in these contexts [40], [41]. Consistent with these reports, our results demonstrate that *DLP* expression is elevated by H_2_O_2_ and UV exposure. Moreover, the apoptosis induced by these insults is reduced dramatically in *DLP* mutants. In contrast to oxidative stress or UV irradiation, γ-ray irradiation (40 gray)-induced apoptosis was unaffected by *DLP* deficiency (data not shown). This is consistent with a previous report, which demonstrated that *DLP* is not associated with radiosensitivity [45]. These findings suggest DLP does not function as a general pro-apoptotic factor, but rather exerts a pro-apoptotic function in response to specific insults, including oxidative stress and UV irradiation. Moreover, the level of DLP in neurons was associated with fly survival rates under oxidative stress conditions. The pan-neuronal overexpression of *DLP* rendered flies more sensitive to oxidative stress than controls, while knockdown or loss of *DLP* resulted in resistance to oxidative stress. Therefore, we believe DLP functions as a stress response mediator that generates appropriate cellular responses to oxidative stress. The similarities between the functions of DLP and Daxx suggest that this oxidative stress response pathway is highly conserved from insects to mammals.

Due to the pronounced increase in oxidative damage within *DJ-1β* mutants [52], we hypothesized that DLP functions as an important mediator of hypersensitivity to oxidative stress in *DJ-1β* mutants. Indeed, DLP expression and translocation from the nucleus to the cytoplasm increased in *DJ-1β* mutants, and *DLP* deficiency almost completely rescued the phenotypes of *DJ-1β* mutants, including oxidative stress-induced DA neuronal loss. Moreover, overexpression of *DJ-1β* reduced the level of endogenous DLP. These findings suggest DLP plays an important function in the oxidative stress-related phenotypes of *DJ-1β* mutant flies and that DJ-1β protects flies against oxidative stress, at least in part, by suppression of DLP expression and cytosolic localization.

These observations raised the question of how DJ-1β negatively regulates *DLP* expression at the transcriptional level under oxidative stress conditions. Our data indicate that DJ-1β controls *DLP* gene expression by regulating the activity of dFOXO. Furthermore, *DLP* harbors a consensus FRE in its promoter region and *dFOXO* overexpression increased *DLP* expression in neurons. Previous studies as well as this work identified DJ-1 as a positive regulator of the PI3K/Akt pathway [19], [49], [50], which suppresses the activity of FOXO by phosphorylation [58]. Therefore, it was not surprising to see that loss of *DJ-1β* function reduced dAkt activity and increased the transcriptional activity of dFOXO (Figure 6G–6H). FOXO activity may be crucial for setting the sensitivity threshold for oxidative stress and determining the appropriate level of stress responses, which ultimately determines whether the cells live or die. From this perspective, the elevated level of dFOXO activity in *DJ-1β* mutants may render their neurons more sensitive to stress, and thus neurons in mutant animals die more readily than their wild-type counterparts. We also found that dFOXO performs a dual role in *DJ-1β* mutant flies, as it is required for the upregulation of *DLP* and is an effector of DLP. Both of these roles increase the DLP-mediated apoptosis in response to oxidative stress in *DJ-1β* mutants. This suggests that dFOXO is involved in the loss of neurons due to oxidative stress, and possibly, *DJ-1* mutation-associated familial PD cases.

In addition to transcriptional regulation, DJ-1 controls DLP translocation from the nucleus to the cytosol. Daxx is translocated to the cytosol under oxidative stress conditions [59] and this translocation is important for its pro-apoptotic function [60]. These studies and our results showed that both mammalian and *Drosophila* DJ-1 strongly suppress the cytosolic translocation of Daxx/DLP. The molecular mechanism by which DJ-1 suppresses the DLP translocation is elusive. It has been proposed that mammalian DJ-1 directly binds to Daxx and inhibits its translocation. However, we did not observe prominent binding between *Drosophila* DJ-1 and DLP (data not shown), suggesting *Drosophila* DJ-1 may regulate the DLP translocation by an alternative mechanism. Interestingly, the mitochondrial targeted DJ-1β efficiently inhibited the Daxx translocation under oxidative stress conditions, suggesting the function of DJ-1 in mitochondria is important for the translocation. Further studies are necessary to understand how DJ-1 inhibits the cytosolic localization of Daxx/DLP under oxidative stress.

**Figure 7:**
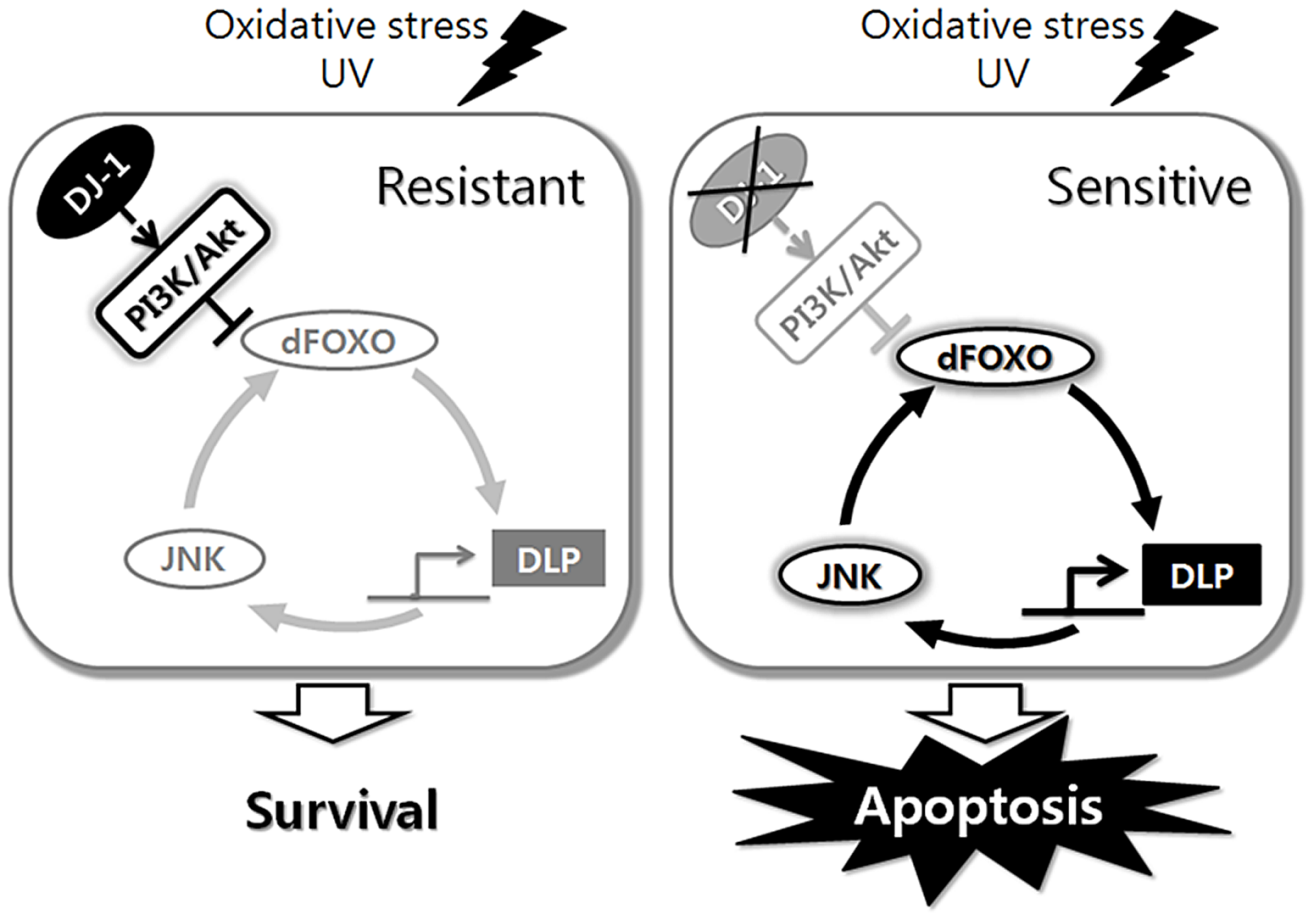
Schematic representation of the role of *Drosophila* DJ-1 in the cellular response to oxidative stress or UV. dFOXO, DLP, and JNK form a circuit that controls cellular responses to stress, and DJ-1 sets neural sensitivity to stress by regulating this circuit.

Our work with DJ-1β and its downstream effector, DLP, has led us to propose the models illustrated in Figure 7. In a wild-type animal, DJ-1 protects the cells from oxidative stress-induced apoptosis. This protection is a result of activation of the PI3K/Akt pathway that inhibits dFOXO. dFOXO, among its many functions, induces *DLP* transcription. *DLP* expression levels have a direct positive correlation with the likelihood of a cell to undergo apoptosis in response to oxidative stress. It is important to note that not only does DJ-1β suppress *DLP* expression, but DJ-1β also prevents DLP translocation to the cytosol, which may be critical for the pro-apoptotic function of DLP. However, once cells are damaged by oxidative stress and UV irradiation, the DLP protein acts through the JNK pathway to initiate apoptosis. Since the JNK pathway can increase dFOXO activity, *DLP* expression can be further increased by a hypothetical feed-forward loop of DLP-JNK-dFOXO. In wild-type animals, this pro-apoptotic loop can be negatively regulated by DJ-1β, while in *DJ-1β* mutant animals, the inability to control this process leads to increased DLP levels and apoptosis. This increased chance of apoptosis may be an important factor in the development of PD.

## Materials and Methods

### *Drosophila* strains

*DJ-1β^ex54^* and *p53^E4^* were previously described [24], [61], and *UAS*-*HA*-*DJ-1β* was generated via microinjection of the corresponding plasmid into *w^1118^* embryos. *EY09290*, *KG01694*, *basket^1^* (*bsk^1^*), *UAS*-*DIAP1*, *UAS*-*dFOXO*, *UAS*-*PTEN*, *elav*-*GAL4*, *Glass multimer reporter* (*GMR*)*-GAL4, tubulin* (*tub*)-*GAL4*, *eyeless* (*ey*)-*GAL4*, *sevenless* (*sev*)-*GAL4*, *wingless* (*wg*)-*lacZ*, and *engrailed* (*en*)-*lacZ* were acquired from the Bloomington *Drosophila* Stock Center (Bloomington, IN, USA). *UAS*-*DLP*-*RNAi* was obtained from the Vienna *Drosophila* RNAi Center (Vienna, Austria). *UAS*-*basket* (*bsk*) and *UAS*-*hemipterous* (*hep*) were gifts from Dr. M. Mlodzik (EMBL, Germany). *UAS*-*puckerd* (*puc*) and *MS1096*-*GAL4* were generously provided by Dr. M. Peifer (University of North Carolina, Pembroke, NC) and Dr. M. Freeman (MRC Laboratory of Molecular Biology, Cambridge, UK), respectively. *UAS*-*cncC* and *UAS*-*cncC*-*RNAi* were gifts from Dr. D. Bohmann (University of Rochester Medical Center). *DLP^U42^* and *dFOXO^21^* were gifts from Dr. I. M. Boros (University of Szeged, Hungary) and Dr. E. Hafen (University of Zurich, Switzerland), respectively. *hemipterous^1^* (*hep^1^*) was gift from Dr. S. Noselli (CNRS, France). *UAS-hemipterous^CA^* (*hep^CA^*, the constitutively active form of *Drosophila JNKK*) was gift from Dr. K. Mastsumoto (Nagoya University, Japan). All fly strains were maintained at 25°C.

### Microarray

Total RNA was extracted from the heads of hydrogen peroxide-treated wild-type and *DJ-1β* mutant flies using an RNeasy Mini kit (Qiagen) in accordance with the manufacturer's instructions. Total RNA was used as a probe for microarray analyses. GeneChip *Drosophila* Genome 2.0 Arrays for *Drosophila melanogaster* were probed, hybridized, stained, and washed in accordance with the manufacturer's recommendations. Hybridized arrays were scanned using an Affymetrix Command Console, and normalization was conducted using an Affymetrix Expression Console 1.1 (MAS5). These experiments were repeated three times for each sample. We required that the fold change difference between the average of three independent wild-type samples and the average of three independent *DJ-1β* mutant samples exceed 1.5 (p≤0.005).

### Generation of *DLP* mutants

To generate *DLP* mutant flies, we used the *EY09290* and *KG01694* lines (Bloomington, USA) containing the *UAS* in the first exon and first intron of the *DLP* gene, respectively. Two *DLP* mutants, *DLP^1^* and *DLP^2^*, were generated via the imprecise excision of a P-element in the *EY09290* line, and *DLP^3^* and *DLP^4^* were obtained from the *KG01694* line. Additionally, the revertant line, *DLP^rv^*, was also generated via precise excision of the P-element in the *EY09290* line. Among the excision lines, the deletion lines were selected via genomic DNA PCR. Genomic DNA was prepared from each independent line, and PCR was conducted with primer pairs to amplify sequences lying upstream and downstream of the P-element insertion site. The deletion sites of the selected lines were determined by sequencing the PCR products. To isogenize the genetic background, *DLP^1^* and *DLP^2^* were backcrossed with *w^1118^* ten times.

### Immunoblotting

A polyclonal antibody against the C-terminus of *Drosophila* DLP (amino acids 1300–1559) was generated in rabbits via injection of pGEX-fused *DLP*. The specificity of the DLP antibody was verified by immunoblotting and immunohistochemistry using wild-type and *DLP* mutant fly tissues (Figure 3C and Figure S2). Cell and fly lysates were prepared in lysis buffer A (150 ml NaCl, 25 mM Tris, 10% Glycerol, 0.1% NP-40, and 1 mM EDTA). For western blotting, the membranes were probed with anti-DLP (1∶1,000 in Tris-buffered saline with Tween 20 (TBST)), anti-lamin (1∶2,000 in TBST; Developmental Studies Hybridoma Bank (DSHB)), anti-β-tubulin (1∶2,000 in TBST; DSHB), anti-phospho-*Drosophila* Akt (Ser505) (1∶1,000 in TBST; Cell Signaling Technology), anti-Akt (1∶1,000 in TBST; Cell Signaling Technology), anti-dFOXO (1∶2,000 in TBST; Cosmo Bio, Japan), or anti-Actin (1∶2,000 in TBST; DSHB at the University of Iowa) antibodies. Western blot analyses were conducted with standard procedures using horseradish peroxidase-conjugated secondary antibodies (1∶2,000 in TBST; Cell Signaling Technology).

### Nuclear/cytosolic fractionation

To separate nuclear and cytosolic fractions, 15 fly heads were collected and homogenized. Nuclear and cytosolic fractions from the fly heads were isolated with Nuclear Extract Kit (Active Motif) in accordance with the manufacturer's instructions.

### RNA *in situ* hybridization

*In situ* hybridization experiments were conducted using a digoxigenin-labeled RNA probe (Roche Applied Science) in accordance with the manufacturer's instructions. The probe was prepared using the PCR product of *DLP*. Hybridization was conducted at 55°C, and the RNA hybrids were detected with alkaline phosphatase (AP)-conjugated anti-digoxigenin antibody followed by nitro blue tetrazolium (NBT)/5-bromo-4-chloro-3-indolyl-phosphate (BCIP) staining.

### Analysis of *Drosophila* development

One hundred embryos of each genotype were placed on grape juice agar plates. After incubation for 2 days at 25°C, the number of larvae hatched was counted to determine embryonic lethality. These larvae were then transferred to standard media (cornmeal, yeast, molasses, agar) and aged at 25°C in upright standard plastic shell vials. Larvae were maintained under non-crowded conditions with 20 individuals per vial. The numbers of pupae and enclosed adult flies were counted. Experiments were repeated 3 times with 100 flies per genotype.

### UV irradiation

UV irradiation experiments were conducted as previously described with some modifications [48]. In brief, 10 mid-aged pupae were collected, and the pupal shells surrounding the heads were surgically removed. The samples were UV irradiated at 10 mJ/cm^2^ using a UV crosslinker (CL-1000, UV Products). Following irradiation, the pupae were kept in darkness until processing, and the survival rate was determined. Each experiment was repeated more than 5 times. To analyze the effects of UV irradiation on cell death or *DLP* gene expression in the embryos, 0–3 h embryos were UV-irradiated at a dose of 50 mJ/cm^2^.

### Oxidative stress test

The effects of oxidative stress on the survival of the indicated lines were evaluated by feeding with hydrogen peroxide. Fifty 3-day-old male flies of the indicated lines were starved for 6 h and then transferred to vials containing 1% hydrogen peroxide in 5% sucrose solution. Surviving flies were counted semi-diurnally. We carried out each survival experiment at least 5 times with 50 flies per genotype (n≥250). In order to evaluate gene expression and protein levels under oxidative stress conditions, the flies were fed with 1% hydrogen peroxide for 3 days.

### Preparation of RNA and real-time quantitative PCR

For real-time quantitative PCR, total RNA from the 20 fly heads was isolated with an RNeasy Protect Mini kit (Qiagen). Then, cDNA was synthesized with a Maxime kit (iNtRON Biotechnology), and real-time quantitative PCR was undertaken using SYBR Green PCR Master Mix (Applied Biosystems) according to the manufacturer's recommended protocols. Real-time quantitative PCR was performed using StepOne Real-time PCR system (Applied Biosystems). Quantification was performed using the ‘delta-delta Ct’ method to normalize to *Actin* transcript levels and to control. Each experiment was repeated at least 5 times (n≥5). The relative level of *DLP*, *d4E-BP* or *dFOXO* mRNA to *Actin* mRNA was statistically analyzed by Student's *t*-test.

### Primers

To determine the deletion sites of the *DLP* mutant lines, PCR was conducted using the following primer pairs: 5′-ACTGCAAATAGTGAATTAAGGCAAC-3′ and 5′- TGCAACATGGGAAGTCTCTG-3′. To quantify the level of gene expression, real-time quantitative PCR was conducted using the following primer pairs: *DLP*, 5′-CACATCCCCAGTGGAATCAC-3′ and 5′-TGCCAACATTGATCTGCTTC-3′; *Actin*, 5′-CACCGGTATCGTTCTGGACT-3′ and 5′-GCGGTGGTGGTGAAAGAGTA-3′; *dFOXO*, 5′-GCCTGGAGGTGCTCAATAAC-3′ and 5′-GTGGCCAGCGGTATATTGAT-3′; and *d4E-BP*, 5′-CCATGATCACCAGGAAGGTT-3′ and 5′-GAAAGCCCGCTCGTAGATAA-3′.

### Terminal deoxynucleotidyl transferase-mediated dUTP-biotin nick end-labeling (TUNEL) assay

For the TUNEL assay, embryos treated with UV were fixed in 4% paraformaldehyde in phosphate-buffered saline (PBS) for 30 min at room temperature. The samples were then washed with PBS and permeabilized by a 2-min incubation in PBS containing proteinase K (10 µg/mL) and 0.1% Triton X-100 on ice. After extensive washing, the samples were incubated for an additional 3 h in TUNEL reaction solution (Roche Applied Science) at 37°C in accordance with the manufacturer's recommendations. After three rinses with PBS, the embryos were incubated with anti-digoxigenin-AP antibody and stained with NBT/BCIP.

### Construction of subcellular organelle-targeted DJ-1β

Full-length *DJ-1β* cDNA was cloned into dsRed2-Mito vector (Clontech) to target DJ-1β to mitochondria. dsRed2-Mito vector contains the mitochondrial targeting sequence from subunit VIII of human cytochrome c oxidase at the N-terminus. For the nucleus-targeted DJ-1β, pEF/3×*NLS*/*Myc* vector (Invitrogen) was used. To construct the cytoplasmic membrane-targeted DJ-1β, 20-amino acid farnesylation signal from c-Ha-Ras was fused to the C-terminus of DJ-1β resulting in pcDNA3 3×*HA DJ-1β-F*. For Golgi-targeted DJ-1β, Golgi targeting sequence from *β* 1,4-galactosyltransferase was fused to the N-terminus of DJ-1β resulting in pcDNA3 Golgi *DJ-1β*.

### Mammalian cell culture and DNA transfection

HeLa cells were grown in DMEM (Invitrogen) supplemented with 10% fetal bovine serum (Invitrogen) at 37°C in a humidified atmosphere of 5% CO_2_. WT and *DJ-1* null SN4741 cells were established from the substantia nigra region of E13.5 wild-type and *DJ-1* knockout mouse embryos, respectively [62]. SN4741 cells were grown in RF medium containing DMEM supplemented with 10% fetal bovine serum, 1% glucose, and L-glutamine (2 mM) at 33°C with 5% CO_2_. The transfection of expression plasmids was performed using Lipofectamine plus reagent (Invitrogen), or PEI (Polyethylenimine, Sigma) according to the manufacturer's instruction.

### Immunocytochemistry

For immunocytochemistry, SN4741 or HeLa cells were sub-cultured on 12-well culture plates coated with poly-L-lysine (Sigma). Appropriately treated cells were washed once with PBS and fixed in 2% paraformaldehyde for 15 min, followed by permeabilization with 0.5% Triton X-100 in PBS for 5 min. Then, the cells were washed with 0.1% Triton X-100 in PBS (PBS-T) and incubated in blocking solution (4% BSA and 1% normal goat serum in PBS-T) for 1 h. Primary antibodies were added to the blocking solution and the cells were incubated overnight at 4°C. After washing with PBS-T 3 times, the cells were incubated with appropriate secondary antibodies in blocking solution for 45 min at room temperature. The antibody-labeled cells were washed with PBS-T 6 times and mounted with mounting solution [100 mg/mL 1,4-diazabicyclo[2.2.2]octane (DABCO) in 90% glycerol]. The slides were observed with LSM710 laser-scanning confocal microscope (Carl Zeiss). All immunostaining experiments with HeLa cells were conducted at least 3 times (n = 300). Anti-mouse DJ-1β [24] and anti-rabbit Daxx (Cell Signaling Technology) were used as primary antibodies. MitoTracker Red CMXRos (Invitrogen) was used to visualize mitochondria.

### Immunohistochemistry

For immunohistochemistry, the wing or eye imaginal discs or adult brains were fixed in 4% paraformaldehyde in PBS at room temperature. The tissues were then washed in PBT (PBS+0.5% Triton X-100) and blocked in PBT with 2% normal goat serum (NGS). The samples were incubated first with rabbit anti-phospho-JNK antibody (1∶200 in PBT containing 2% NGS; Promega) or rabbit anti-phospho-histone H3 antibody (1∶200 in PBT containing 2% NGS; Upstate Biotechnology) or rabbit anti-DLP antibody (1∶200 in PBT containing 2% NGS) or rabbit anti-tyrosine hydroxylase antibody (1∶50 in PBT containing 2% NGS; Pel-Freez Biologicals) and then subsequently incubated with rhodamine-labeled goat anti-rabbit immunoglobulin G secondary antibody (1∶200 in PBT; Sigma-Aldrich).

### Ectopic gene expression with the *UAS-GAL4* system

The *UAS-GAL4* system was used to evaluate the phenotypes induced by the overexpression of several target genes, including *DLP*. The *GAL4* gene was placed near a tissue-specific enhancer, allowing for the ectopic expression of the target gene in the desired tissue. *GMR-GAL4*, *MS1096*-*GAL4*, *elav*-*GAL4*, *tub*-*GAL4*, and *ey*-*GAL4* were used to induce target gene expression in the eye, the whole wing, the nervous system, the whole body, and the eye, respectively.

### Acridine orange staining

Acridine orange staining was conducted as previously described [63] with some modifications. The wing or eye imaginal discs of stage L3 larvae were dissected in PBS. In order to characterize the effects of oxidative stress on cell death, we incubated the larval brains for 24 h in Schneider's *Drosophila* media with 0.1% hydrogen peroxide. The discs or brains were then incubated for 5 min in 1.6×10^−6^ M acridine orange (Sigma-Aldrich) and briefly rinsed in PBS. The samples were subsequently observed under an Axiophot2 fluorescence microscope (Carl Zeiss).

### 5-bromo-4-chloro-3-indolyl-β-D-galactopyranoside (X-gal) staining

For X-gal staining, the wing discs were fixed for 4 min in 4% formaldehyde in PBS, washed, and incubated in standard X-gal staining solution (4.9 mM X-gal, 3.1 mM K_4_Fe(CN)_6_, 3.1 mM K_3_Fe(CN)_6_, 1 mM MgCl_2_, 150 mM NaCl, 10 mM Na_2_HPO_4_, 10 mM NaH_2_PO_4_, 0.3% Triton X-100) for 30 min at 37°C before observation.

### S2 cell culture and luciferase assay

*Drosophila* S2 cells were transiently transfected using the Effectene transfection reagent (Qiagen Inc., Valencia, CA) using a standard protocol. After 24 h, CuSO_4_ (Sigma) was added to a final concentration of 0.6 mM for the optimal expression of the dFOXO A3 construct (a gift from Dr. Oscar Puig, Roche). Dual luciferase assays were performed using the dual luciferase assay system (Promega Corp., Madison, WI). Harvested cells were lysed with luciferase cell lysis buffer. Cell lysates (20 µl out of 30 µl total lysate per sample) were analyzed for firefly luciferase activity by adding 20 µl of firefly reaction buffer. Furthermore, *Renilla* luciferase activity was measured by adding 20 µl of *Renilla* reaction buffer. Luminescence was measured from a 96-well plate by using VictorX5 multilabel plate reader (Perkin-Elmer). The data are presented as fold changes relative to the negative control (transfection with the pMT empty vector as an effecter plasmid) normalized to 1.

### Climbing assay

The climbing assay was conducted as previously described [64], [65] with some modifications. Ten male flies of the indicated lines were transferred into the climbing ability test vial and incubated for 1 h at room temperature for environmental acclimation. After tapping the flies down to the bottom, we counted the number of flies that climbed to the top of the vial within 4 sec. Ten trials were conducted for each group. The experiment was repeated at least 10 times with independently derived transgenic lines. Climbing scores (ratio of the number of flies that climbed to the top to the total number of flies, expressed as a percentage) were obtained for each test group, and the mean climbing score for 10 repeated tests was compared to the scores of the wild-type flies. All climbing assay experiments were conducted at 25°C.

### Statistics

Western blotting data were measured using the Multi gauge V3.1 (Fuji, Japan) software program and converted into ratios of band intensity relative to the controls. Using the non-parametric Wilcoxon signed-rank test or the Kruskal-Wallis test, the data were analyzed to detect any statistical differences between treatments. In particular, when the data analyzed with the Kruskal-Wallis test revealed a statistical difference, the data were arcsine-transformed and subsequently analyzed by ANOVA followed by Tukey's HSD *post-hoc* analysis. The climbing assay data were arcsine-transformed, and then ANOVA with Tukey's HSD *post hoc* analyses were conducted to detect any differences in climbing ability between treatments. The Kaplan-Meier estimator and the log-rank test were conducted on the pooled cumulative survival data to determine whether each treatment had any effect on the longevity of individuals using Online Application Survival Analysis Lifespan Assays (http://sbi.postech.ac.kr/oasis) [66].

## Supporting Information

Figure S1 DA neurons in the brains of wild-type and *DJ-1β* mutant flies grown under standard laboratory condition. (A) DA neurons visualized by immunohistochemical analysis with anti-tyrosine hydroxylase antibody in the brains of wild-type (WT) and *DJ-1β* mutant (*DJ-1β^ex54^*) flies fed cornmeal-soybean standard fly food. The lower pictures, including DM, DL1, DL2, and PM, are the magnified areas of the upper pictures. Magnification of the upper pictures, 100×; Magnification of the lower pictures, 400×. (B) Graphs showing the number of DA neurons in each cluster of WT and *DJ-1β^ex54^* flies (n = 10). No significant difference is observed (Student *t*-test). The data are expressed as mean ± s.e. values. (C) Acridine orange staining of the larval brains of WT control and *DJ-1β^ex54^*. DM, dorsomedial clusters; DL, dorsolateral clusters; PM, posteromedial clusters.
(TIF)

Figure S2 DLP expression in the wild-type, *DLP*-overexpressing, and *DLP*-deficient tissues. (A–B) Confocal micrographs of eye imaginal discs (A) and of the larval brains (B). Micrographs of immunostaining with anti-DLP antibody show DLP expression in the wild-type and *DLP*-overexpressing (*GMR*>*DLP* and *elav*>*DLP*) tissues, but not in the *DLP* mutants (*DLP^1^*). (A) Magnification of left pictures, 200×; Magnification of right pictures, 400×. (B) Magnification of left pictures, 100×; Magnification of right pictures, 400×. The genotypes of the samples are *GMR*>*DLP* (*GMR-GAL4*/*EY09290*) and elav>*DLP* (*EY09290*/+; *elav-GAL4*/+).
(TIF)

Figure S3 Subcellular localization of DLP in the eye imaginal discs of wild-type and *DJ-1β* mutant larvae. (A) Nuclear localization of DLP in the eye imaginal disc of wild-type (WT) larvae. (B) Increased DLP level in the cytosol of the eye imaginal disc of *DJ-1β* mutant (*DJ-1β^ex54^*) larvae. Hoechst staining was used to visualize the nuclei. (A–B) Magnification, 1,600×.
(TIF)

Figure S4 Regulation of Daxx translocation by *Drosophila* DJ-1 in mammalian cells. (A) Confocal images showing the subcellular localization of Daxx in *DJ-1* null SN4741 cells transfected with wild-type *DJ-1β* or nucleus (Nuc)-, cytoplasmic membrane (CM)-, Golgi (Golgi)-, or mitochondria (Mito)- targeted DJ-1β. The cells were treated with 0.4 mM H_2_O_2_ for 1 h. Hoechst-stained regions represent nuclei. Daxx was expressed in blue in merged images. (B) Confocal images showing subcellular localization of Daxx in HeLa cells. Translocation of Daxx from the nucleus to the cytosol was induced by 1 mM H_2_O_2_ treatment for 2 h. MitoTracker-stained spots represent mitochondria. (C) Confocal images showing subcellular localization of Daxx in HeLa cells transfected with wild-type DJ-1β or mitochondrial targeted DJ-1β (Mito-DJ-1β). The cells were treated with 1 mM H_2_O_2_ for 2 h. MitoTracker-stained spots represent mitochondria. Daxx was expressed in blue in merged images.
(TIF)

Figure S5 Survival rates of *DLP* mutants under oxidative stress conditions. (A–B) *DLP* loss-of-function mutants (A, *DLP^3^* and *DLP^4^*) and *DLP trans*-heterozygous mutants (B, *DLP^1^*/*DLP^U42^*) were resistant to H_2_O_2_ treatment relative to the wild-type (WT) strain (log-rank test: n≥250, p<0.01).
(TIF)

Figure S6 The effects of *DLP* overexpression on various biological processes. (A) Confirmation of ectopic *DLP* gene expression in wing imaginal discs via RNA *in situ* hybridization. *DLP*-overexpressing wing (*MS1096*>*DLP*), but not the control (*MS1096*/Y), evidenced a strong *DLP* mRNA signal. (B–C) Survival rates (B) and climbing ability (C) of flies pan-neuronally overexpressing *DLP*. Pan-neuronal overexpression of *DLP* (*elav>DLP*) reduced both the survival rate of the embryos (ANOVA: n≥10, p<0.01) (B) and climbing ability (Wilcoxon rank sum test: n = 30, p<0.01) (C) relative to the controls (*elav*/Y). All data are expressed as means ± s.e. values. (D–E) Immunostaining using anti-phospho-histone H3 antibody (D) and X-gal staining (E) of control (*MS1096*/Y, *wg-lacZ*/+, or *en-lacZ*/+) and *DLP*-overexpressing (*MS1096*>*DLP*, *MS1096>DLP*, *wg-lacZ*, or *MS1096>DLP*, *en-lacZ*) wing imaginal discs. *wg*-*lacZ* and *en*-*lacZ* are transgenes expressing *lacZ* under the control of the *wingless* (*wg*) and *engrailed* (*en*) gene promoters, respectively. The genotypes of the samples were *elav*/Y (*elav-GAL4*/Y), *elav>DLP* (*elav-GAL4*/Y; *EY09290*/+), *MS1096*/Y (*MS1096-GAL4*/Y), *MS1096>DLP* (*MS1096-GAL4*/Y; *EY09290*/+), *wg-lacZ*/+ (*MS1096-GAL4*/Y; *wg-lacZ*/+), *MS1096>DLP*, *wg-lacZ* (*MS1096-GAL4*/Y; *EY09290*/*wg-lacZ*), *en-lacZ*/+ (*MS1096-GAL4*/Y; *en-lacZ*/+), and *MS1096>DLP*, *en-lacZ* (*MS1096-GAL4*/Y; *EY09290*/*en-lacZ*). pH3, phospho-histone H3.
(TIF)

Figure S7 *DLP* deficiency reduces the loss of DA neurons in *DJ-1β* mutants under conditions of oxidative stress. DA neurons visualized by immunohistochemical analysis with anti-tyrosine hydroxylase antibody in the brains of wild-type (A, WT), *DJ-1β* mutant (B, *DJ-1β^ex54^*), and double mutant of *DLP* and *DJ-1β* (C, *DLP^1^*; *DJ-1β^ex54^*) flies fed with 1% H_2_O_2_ for 3 days. The lower pictures, including DM, DL1, DL2, and PM, are the magnified areas of the upper pictures. Magnification of the upper pictures, 100×; Magnification of the lower pictures, 400×. DM, dorsomedial clusters; DL, dorsolateral clusters; PM, posteromedial clusters.
(TIF)

Figure S8 Effect of *cncC* or *p53* level on the regulation of DLP protein level. (A) DLP protein levels in the control (*elav*/Y), *cncC*-overexpressing (*elav*>*cncC*) and *cncC* knock-down (*elav*>*cncCi*) fly heads (n = 3). (B) DLP protein levels in the heads of the control (WT), *DJ-1β* mutant (*DJ-1β^ex54^*) and double mutant of *DJ-1β* and *p53* (*DJ-1β^ex54^*, *p53^E4^*) flies fed with 1% H_2_O_2_ for 3 days. Actin was used as an internal control. The genotypes of the samples were *elav*/Y (*elav*-*GAL4*/Y), *elav*>*cncC* (*elav*-*GAL4*/Y;; *UAS*-*cncC*/+), *elav*>*cncCi* (*elav*-*GAL4*/Y; *UAS*-*cncC*-*RNAi*/+), *DJ-1β^ex54^* (*DJ-1β^ex54^*/*DJ-1β^ex54^*), and *DJ-1β^ex54^*, *p53^E4^* (*DJ-1β^ex54^*, *p53^E4^*/*DJ-1β^ex54^*, *p53^E4^*).
(TIF)

Table S1 The list of genes for which expression is up- and down-regulated in *DJ-1β* mutant fly heads compared to wild type under the 1% hydrogen peroxide insulted condition.
(DOCX)
