# Supplementary figures and images for: Drosophila DJ-1 Decreases Neural Sensitivity to Stress by Negatively Regulating Daxx-Like Protein through dFOXO
Source: PLoS Genet. 2013 Apr 4;9(4):e1003412. doi: 10.1371/journal.pgen.1003412 (PMC3616925; doi:10.1371/journal.pgen.1003412)

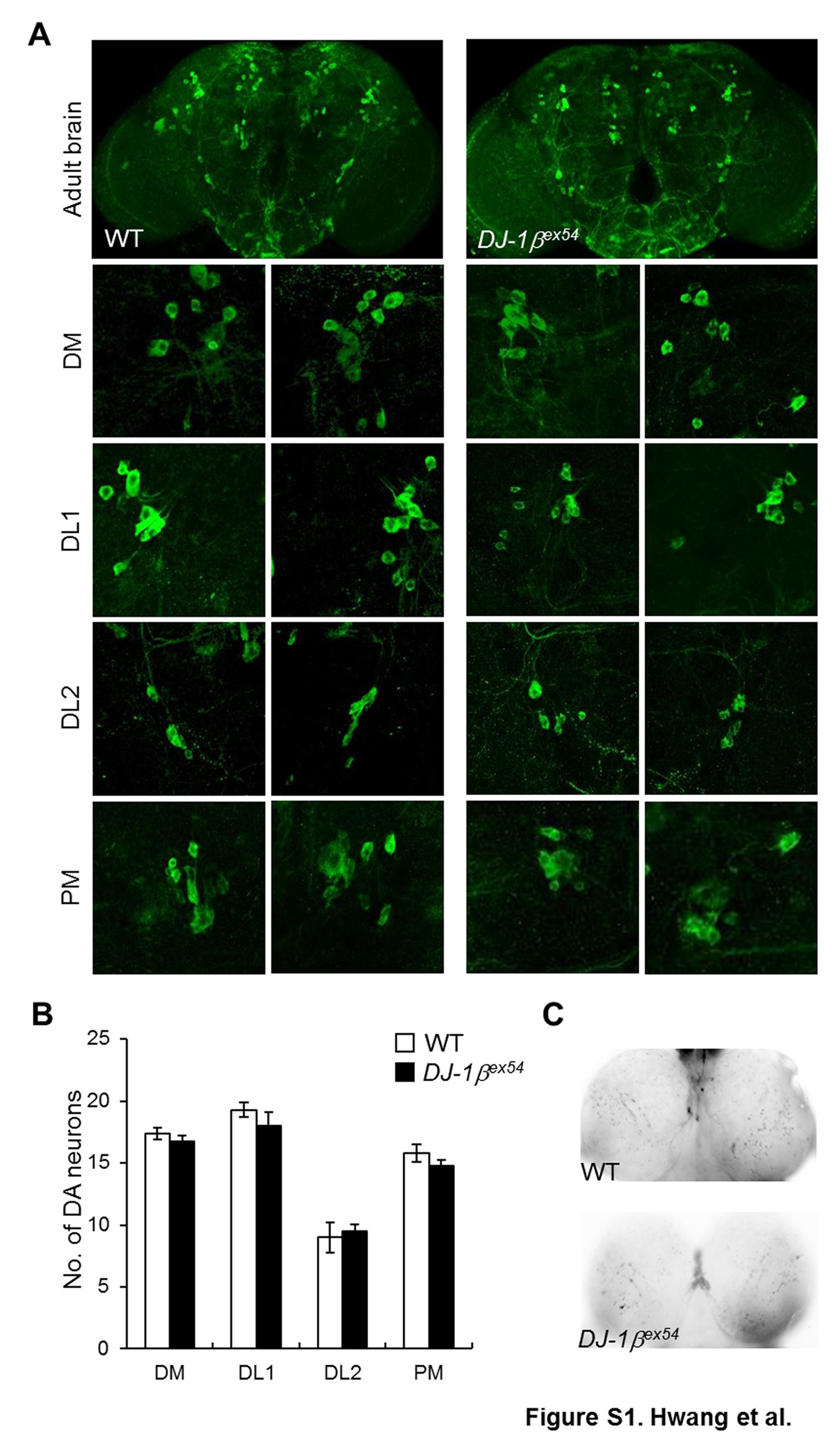

Supplement: Figure S1 — DA neurons in the brains of wild-type and DJ-1β mutant flies grown under standard laboratory condition. (A) DA neurons visualized by immunohistochemical analysis with anti-tyrosine hydroxylase antibody in the brains of wild-type (WT) and DJ-1β mutant (DJ-1βex54) flies fed cornmeal-soybean standard fly food. The lower pictures, including DM, DL1, DL2, and PM, are the magnified areas of the upper pictures. Magnification of the upper pictures, 100×; Magnification of the lower pictures, 400×. (B) Graphs showing the number of DA neurons in each cluster of WT and DJ-1βex54 flies (n = 10). No significant difference is observed (Student t-test). The data are expressed as mean ± s.e. values. (C) Acridine orange staining of the larval brains of WT control and DJ-1βex54. DM, dorsomedial clusters; DL, dorsolateral clusters; PM, posteromedial clusters. (TIF) [file pgen.1003412.s001.tif]

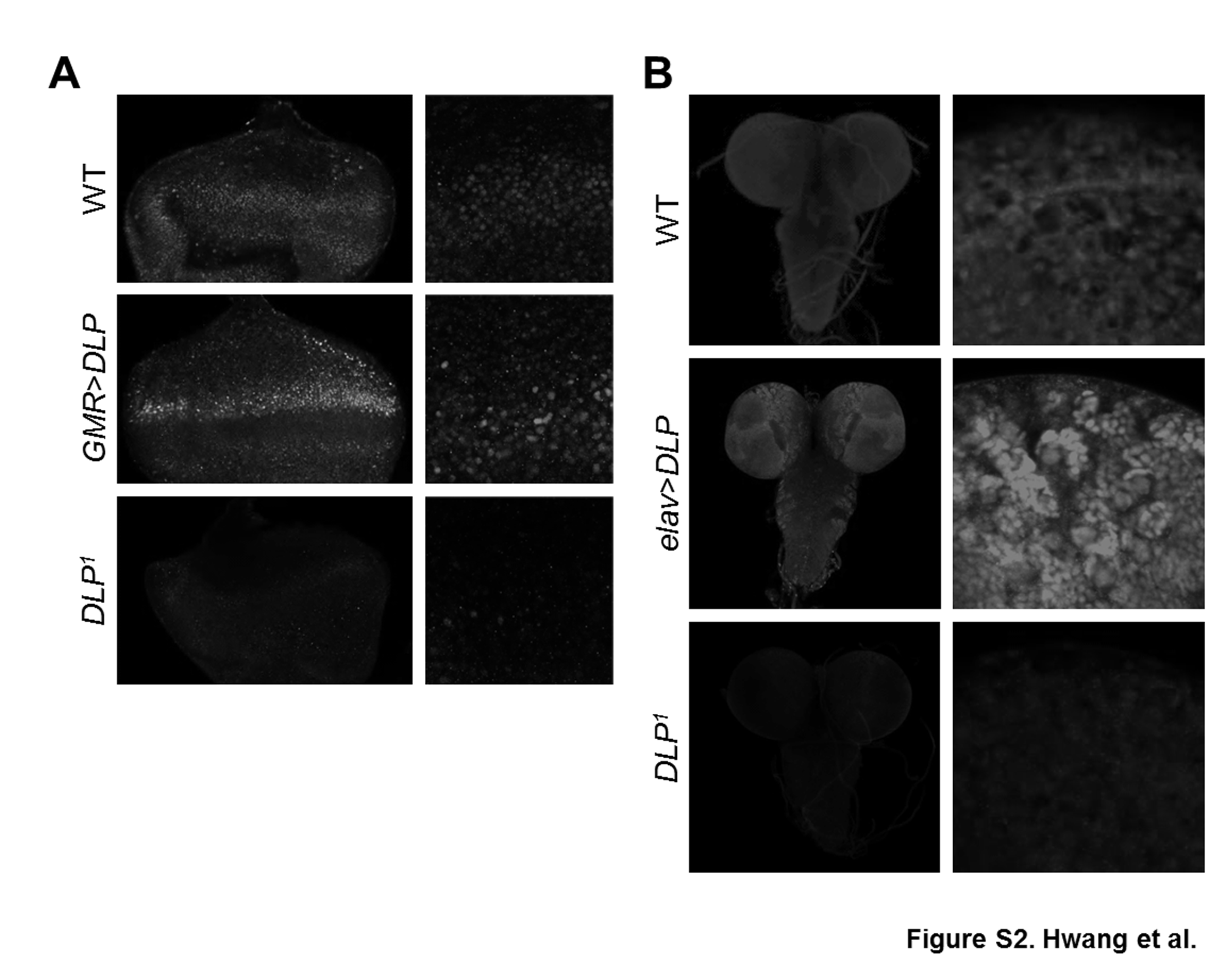

Supplement: Figure S2 — DLP expression in the wild-type, DLP-overexpressing, and DLP-deficient tissues. (A–B) Confocal micrographs of eye imaginal discs (A) and of the larval brains (B). Micrographs of immunostaining with anti-DLP antibody show DLP expression in the wild-type and DLP-overexpressing (GMR>DLP and elav>DLP) tissues, but not in the DLP mutants (DLP1). (A) Magnification of left pictures, 200×; Magnification of right pictures, 400×. (B) Magnification of left pictures, 100×; Magnification of right pictures, 400×. The genotypes of the samples are GMR>DLP (GMR-GAL4/EY09290) and elav>DLP (EY09290/+; elav-GAL4/+). (TIF) [file pgen.1003412.s002.tif]

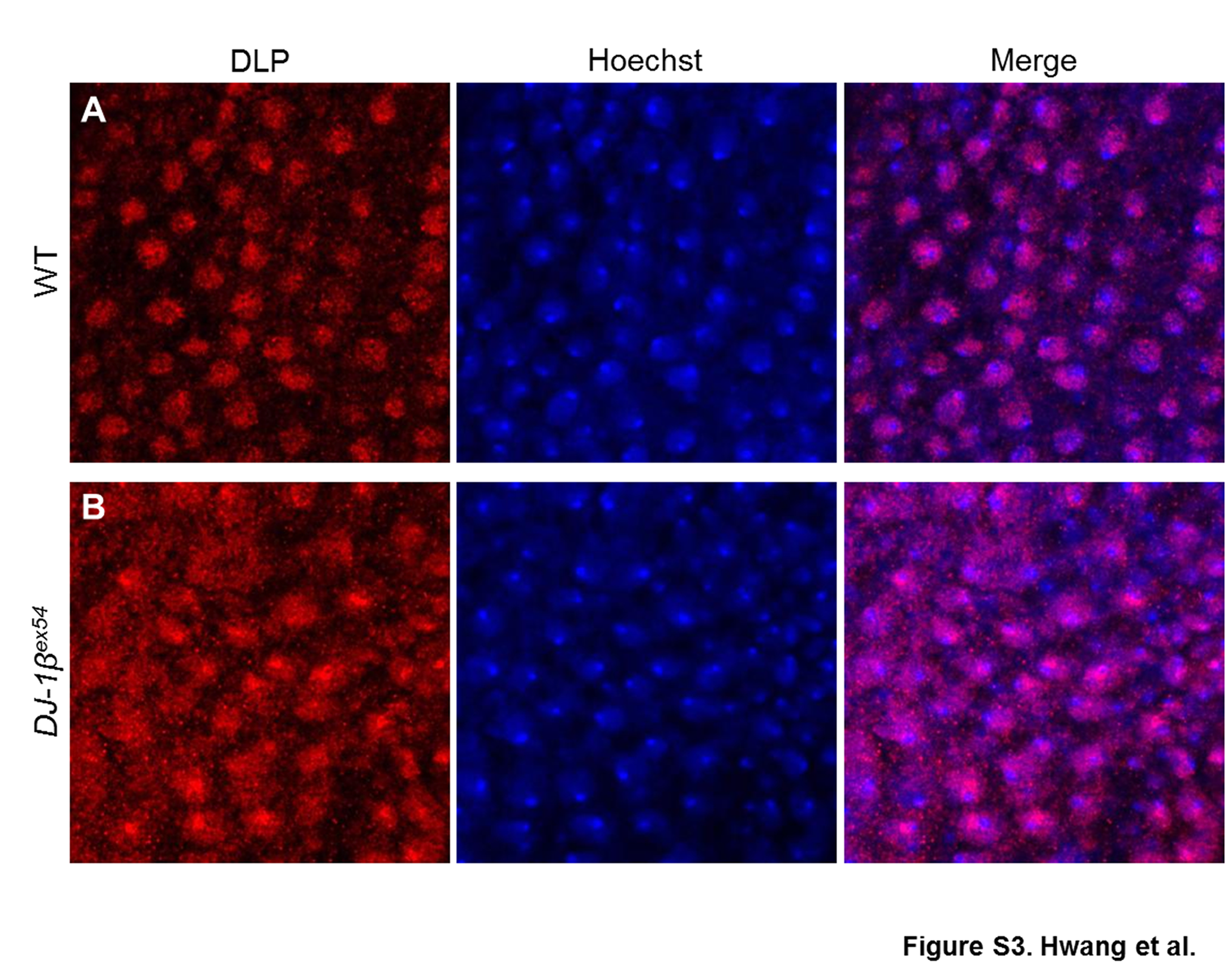

Supplement: Figure S3 — Subcellular localization of DLP in the eye imaginal discs of wild-type and DJ-1β mutant larvae. (A) Nuclear localization of DLP in the eye imaginal disc of wild-type (WT) larvae. (B) Increased DLP level in the cytosol of the eye imaginal disc of DJ-1β mutant (DJ-1βex54) larvae. Hoechst staining was used to visualize the nuclei. (A–B) Magnification, 1,600×. (TIF) [file pgen.1003412.s003.tif]

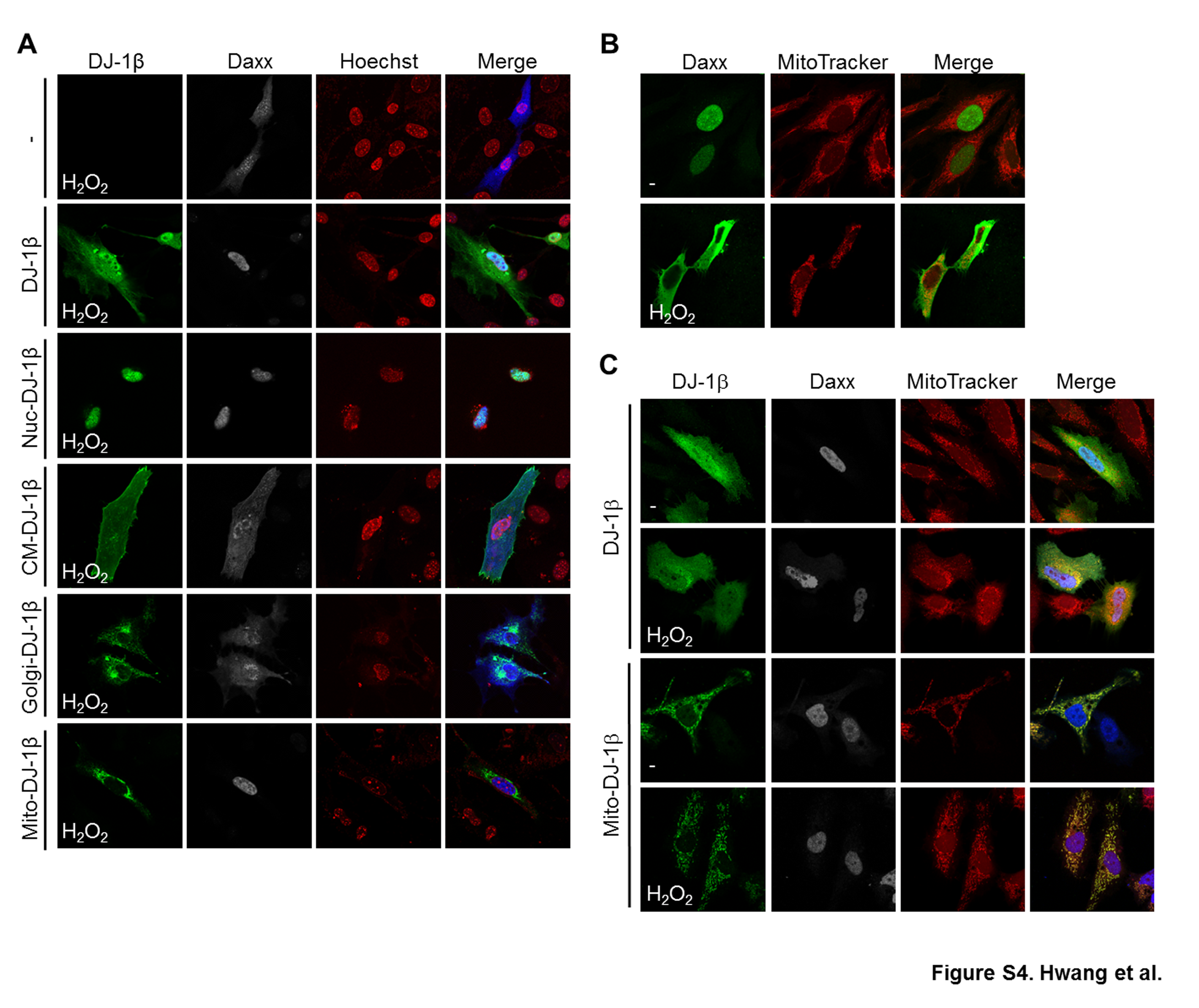

Supplement: Figure S4 — Regulation of Daxx translocation by Drosophila DJ-1 in mammalian cells. (A) Confocal images showing the subcellular localization of Daxx in DJ-1 null SN4741 cells transfected with wild-type DJ-1β or nucleus (Nuc)-, cytoplasmic membrane (CM)-, Golgi (Golgi)-, or mitochondria (Mito)- targeted DJ-1β. The cells were treated with 0.4 mM H2O2 for 1 h. Hoechst-stained regions represent nuclei. Daxx was expressed in blue in merged images. (B) Confocal images showing subcellular localization of Daxx in HeLa cells. Translocation of Daxx from the nucleus to the cytosol was induced by 1 mM H2O2 treatment for 2 h. MitoTracker-stained spots represent mitochondria. (C) Confocal images showing subcellular localization of Daxx in HeLa cells transfected with wild-type DJ-1β or mitochondrial targeted DJ-1β (Mito-DJ-1β). The cells were treated with 1 mM H2O2 for 2 h. MitoTracker-stained spots represent mitochondria. Daxx was expressed in blue in merged images. (TIF) [file pgen.1003412.s004.tif]

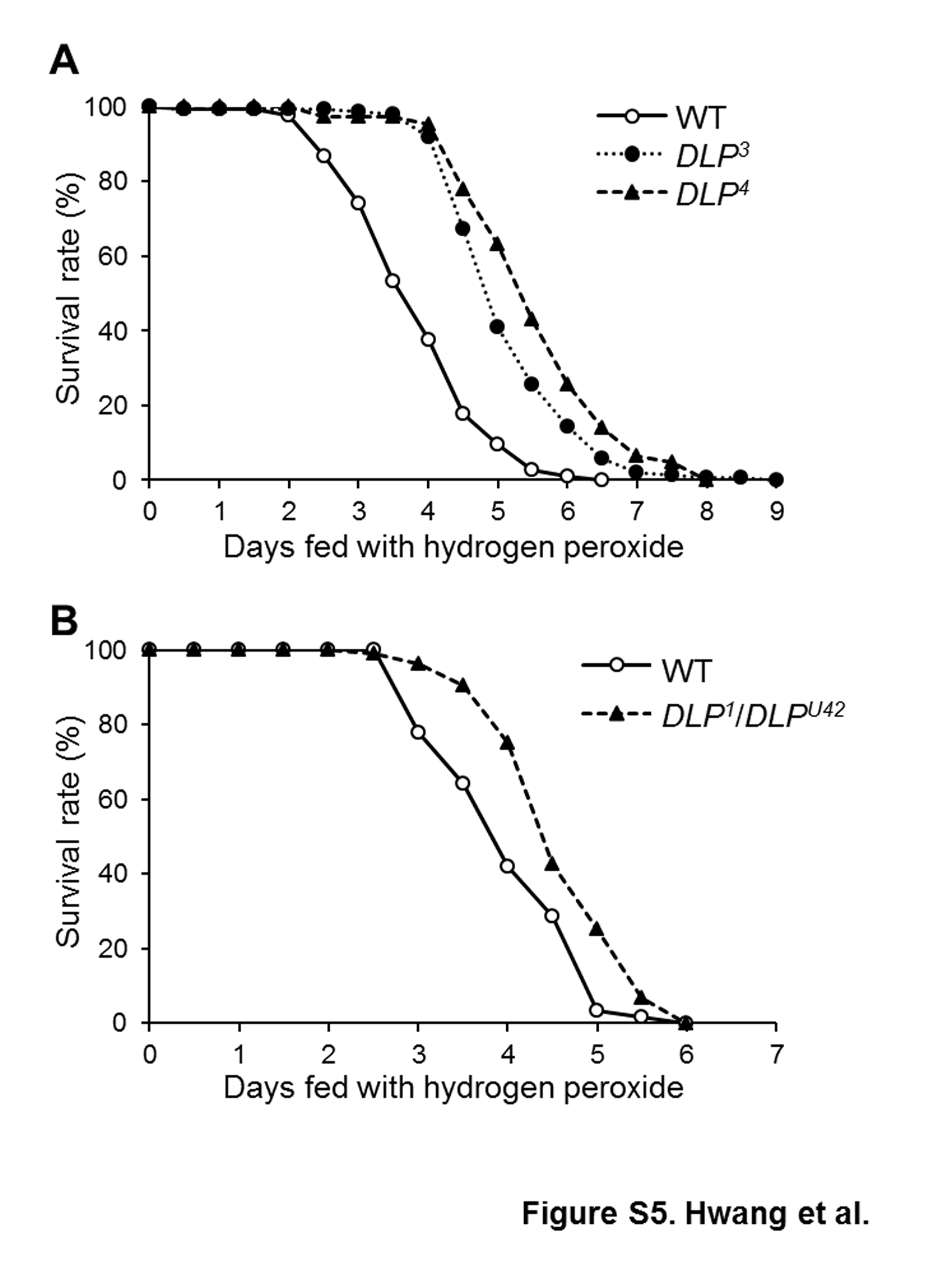

Supplement: Figure S5 — Survival rates of DLP mutants under oxidative stress conditions. (A–B) DLP loss-of-function mutants (A, DLP3 and DLP4) and DLP trans-heterozygous mutants (B, DLP1/DLPU42) were resistant to H2O2 treatment relative to the wild-type (WT) strain (log-rank test: n≥250, p<0.01). (TIF) [file pgen.1003412.s005.tif]

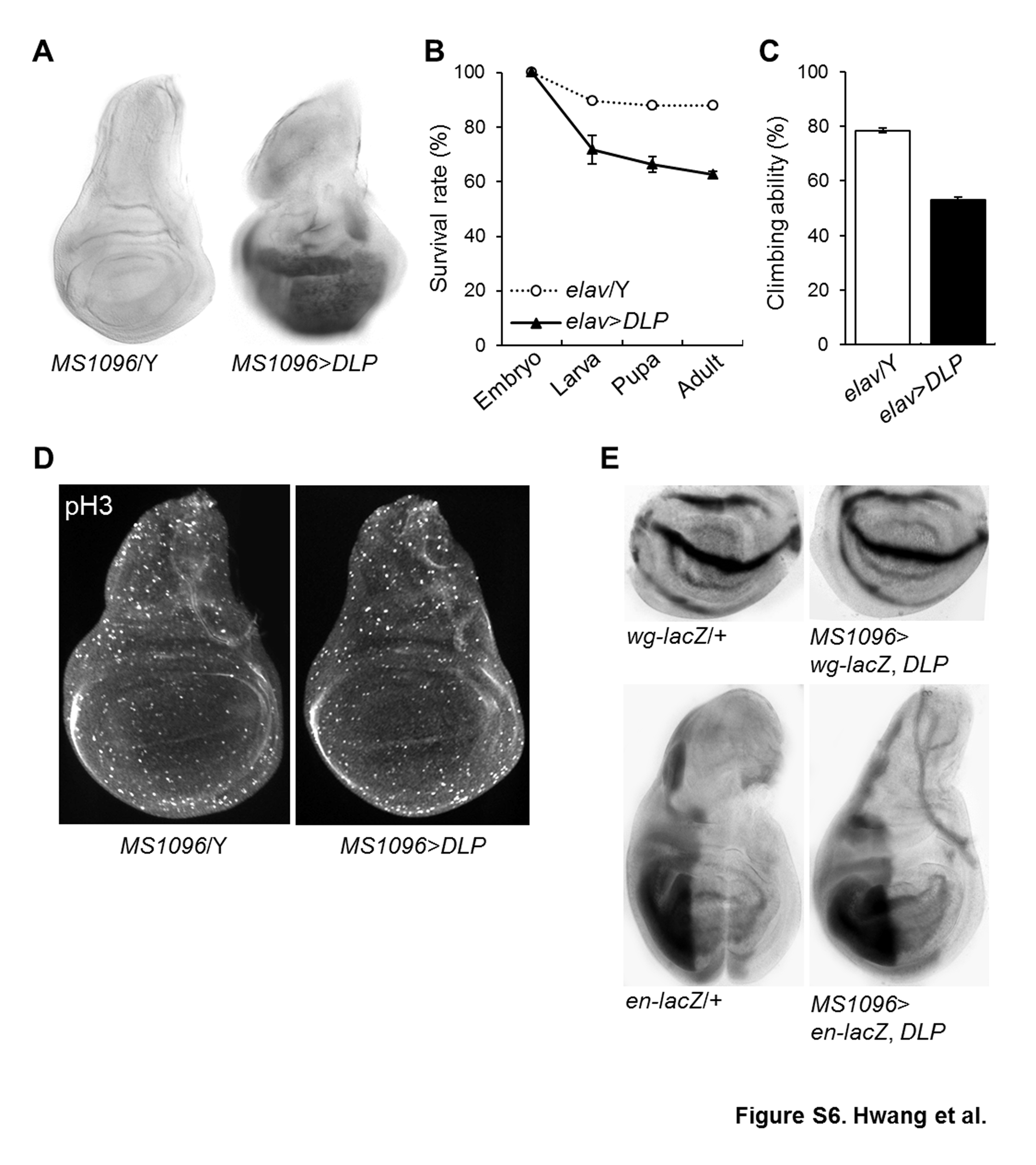

Supplement: Figure S6 — The effects of DLP overexpression on various biological processes. (A) Confirmation of ectopic DLP gene expression in wing imaginal discs via RNA in situ hybridization. DLP-overexpressing wing (MS1096>DLP), but not the control (MS1096/Y), evidenced a strong DLP mRNA signal. (B–C) Survival rates (B) and climbing ability (C) of flies pan-neuronally overexpressing DLP. Pan-neuronal overexpression of DLP (elav>DLP) reduced both the survival rate of the embryos (ANOVA: n≥10, p<0.01) (B) and climbing ability (Wilcoxon rank sum test: n = 30, p<0.01) (C) relative to the controls (elav/Y). All data are expressed as means ± s.e. values. (D–E) Immunostaining using anti-phospho-histone H3 antibody (D) and X-gal staining (E) of control (MS1096/Y, wg-lacZ/+, or en-lacZ/+) and DLP-overexpressing (MS1096>DLP, MS1096>DLP, wg-lacZ, or MS1096>DLP, en-lacZ) wing imaginal discs. wg-lacZ and en-lacZ are transgenes expressing lacZ under the control of the wingless (wg) and engrailed (en) gene promoters, respectively. The genotypes of the samples were elav/Y (elav-GAL4/Y), elav>DLP (elav-GAL4/Y; EY09290/+), MS1096/Y (MS1096-GAL4/Y), MS1096>DLP (MS1096-GAL4/Y; EY09290/+), wg-lacZ/+ (MS1096-GAL4/Y; wg-lacZ/+), MS1096>DLP, wg-lacZ (MS1096-GAL4/Y; EY09290/wg-lacZ), en-lacZ/+ (MS1096-GAL4/Y; en-lacZ/+), and MS1096>DLP, en-lacZ (MS1096-GAL4/Y; EY09290/en-lacZ). pH3, phospho-histone H3. (TIF) [file pgen.1003412.s006.tif]

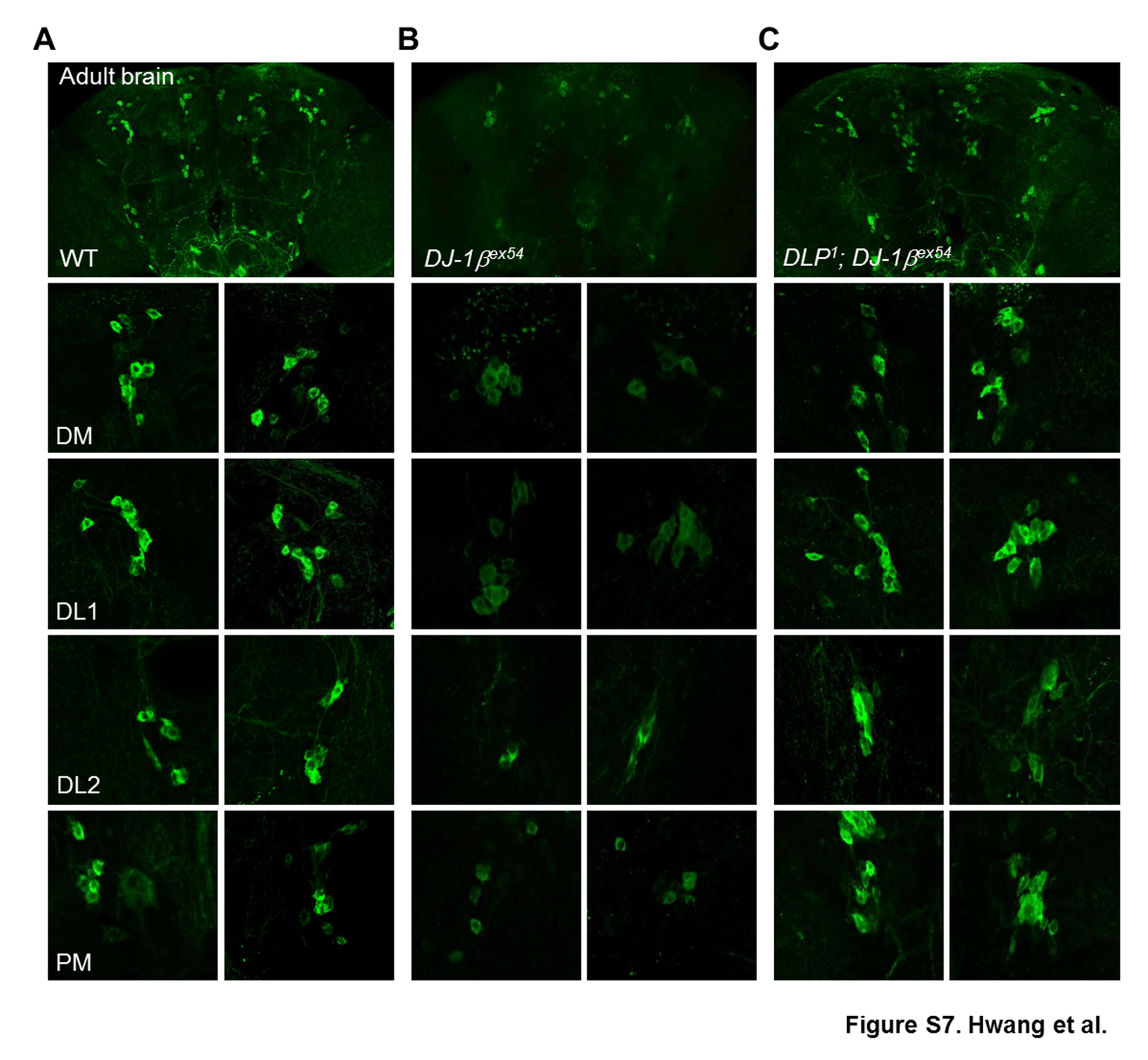

Supplement: Figure S7 — DLP deficiency reduces the loss of DA neurons in DJ-1β mutants under conditions of oxidative stress. DA neurons visualized by immunohistochemical analysis with anti-tyrosine hydroxylase antibody in the brains of wild-type (A, WT), DJ-1β mutant (B, DJ-1βex54), and double mutant of DLP and DJ-1β (C, DLP1; DJ-1βex54) flies fed with 1% H2O2 for 3 days. The lower pictures, including DM, DL1, DL2, and PM, are the magnified areas of the upper pictures. Magnification of the upper pictures, 100×; Magnification of the lower pictures, 400×. DM, dorsomedial clusters; DL, dorsolateral clusters; PM, posteromedial clusters. (TIF) [file pgen.1003412.s007.tif]

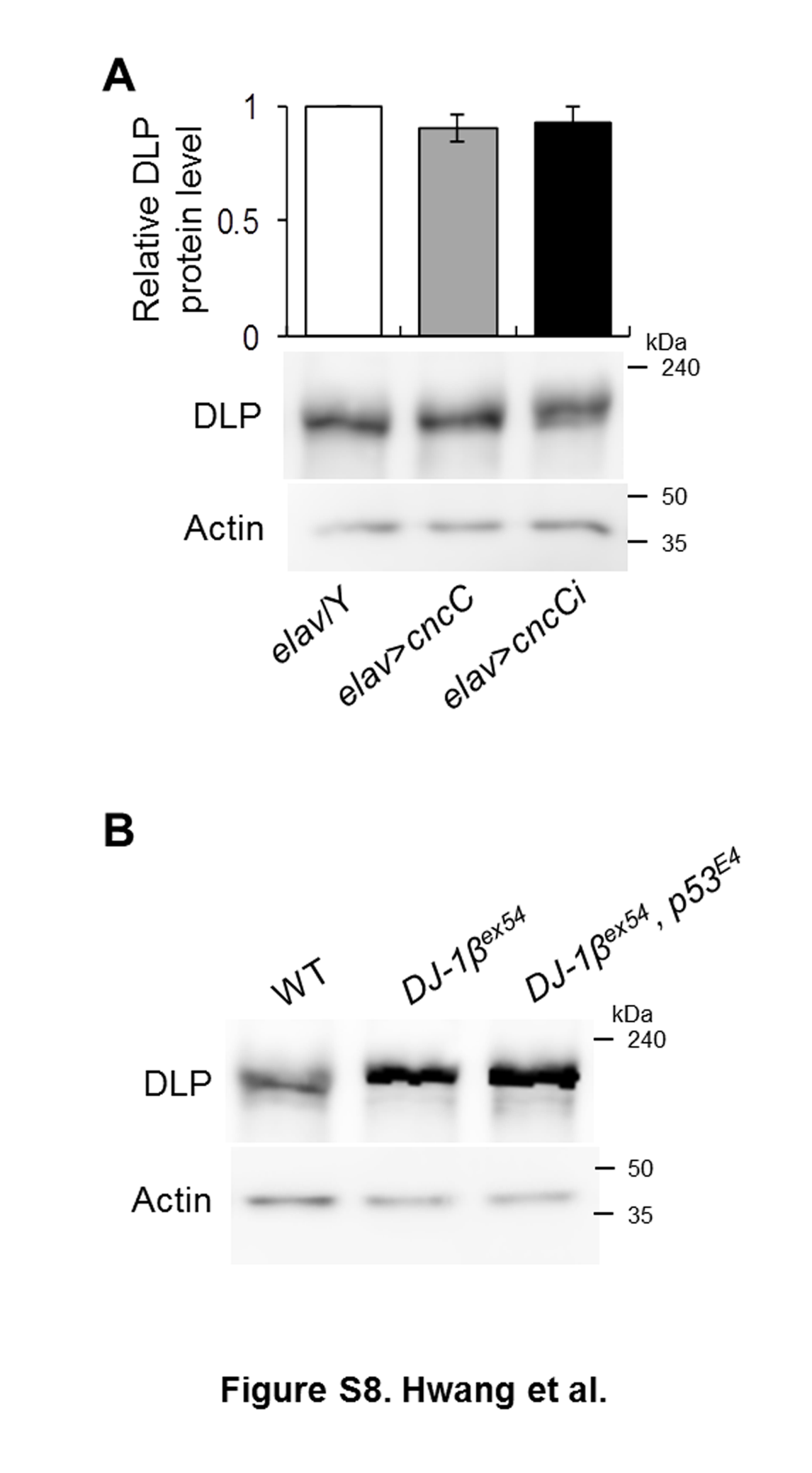

Supplement: Figure S8 — Effect of cncC or p53 level on the regulation of DLP protein level. (A) DLP protein levels in the control (elav/Y), cncC-overexpressing (elav>cncC) and cncC knock-down (elav>cncCi) fly heads (n = 3). (B) DLP protein levels in the heads of the control (WT), DJ-1β mutant (DJ-1βex54) and double mutant of DJ-1β and p53 (DJ-1βex54, p53E4) flies fed with 1% H2O2 for 3 days. Actin was used as an internal control. The genotypes of the samples were elav/Y (elav-GAL4/Y), elav>cncC (elav-GAL4/Y;; UAS-cncC/+), elav>cncCi (elav-GAL4/Y; UAS-cncC-RNAi/+), DJ-1βex54 (DJ-1βex54/DJ-1βex54), and DJ-1βex54, p53E4 (DJ-1βex54, p53E4/DJ-1βex54, p53E4). (TIF) [file pgen.1003412.s008.tif]
