# Supplementary material for: Drosophila DJ-1 Decreases Neural Sensitivity to Stress by Negatively Regulating Daxx-Like Protein through dFOXO
Source: PLoS Genet. 2013 Apr 4;9(4):e1003412. doi: 10.1371/journal.pgen.1003412 (PMC3616925; doi:10.1371/journal.pgen.1003412)
Supplement: Table S1 — The list of genes for which expression is up- and down-regulated in DJ-1β mutant fly heads compared to wild type under the 1% hydrogen peroxide insulted condition. (DOCX) [file pgen.1003412.s009.docx]

Table. S1. Hwang et al.

| Gene Name | Flybase ID | log2 ratio | p-value |
| --- | --- | --- | --- |
| **Up-regulated genes** |  |  |  |
| ***Metabolic process*** |  |  |  |
| CG10352 | FBgn0030348 | 0.95 | 0.00455 |
| CG10399 | FBgn0031877 | 0.98 | 0.00376 |
| CG10463 | FBgn0032819 | 1.59 | 0.00225 |
| CG12512 | FBgn0031703 | 2.05 | 0.00179 |
| CG14721 | FBgn0037942 | 0.62 | 0.00177 |
| CG14934 | FBgn0032381 | 2.45 | 0.00208 |
| CG15313 | FBgn0030173 | 1.28 | 0.00244 |
| CG4279 | FBgn0034600 | 0.72 | 0.00301 |
| CG5599 | FBgn0030612 | 0.61 | 0.00154 |
| CG6113 | FBgn0032264 | 1.02 | 0.00401 |
| CG7367 | FBgn0031976 | 1.45 | 0.00324 |
| CG7433 | FBgn0036927 | 2.18 | 0.00429 |
| CG9344 | FBgn0034564 | 2.26 | 0.00096 |
| CHIP | FBgn0027052 | 0.73 | 0.00158 |
| Lipase 1 | FBgn0023496 | 0.61 | 0.00362 |
| meiotic recombination 11 | FBgn0020270 | 0.97 | 0.00293 |
| tafazzins | FBgn0026619 | 0.67 | 0.00163 |
| Tyrosine decarboxylase 1 | FBgn0050445 | 0.88 | 0.00358 |
| ***Oxidative stress*** |  |  |  |
| CG10638 | FBgn0036290 | 1.36 | 0.00099 |
| CG1236 | FBgn0037370 | 0.71 | 0.00301 |
| CG12466 | FBgn0069972 | 1.55 | 0.00004 |
| CG12466 | FBgn0069972 | 0.86 | 0.00001 |
| CG40485 | FBgn0069973 | 5.35 | 0.00028 |
| CG6638 | FBgn0035911 | 0.60 | 0.00159 |
| Dihydrofolate reductase | FBgn0004087 | 1.19 | 0.00196 |
| dynein-related heavy chain polypeptide | FBgn0013810 | 0.80 | 0.00124 |
| Glutathione S transferase E3 | FBgn0063497 | 0.82 | 0.00300 |
| Glutathione S transferase E4 | FBgn0063496 | 0.99 | 0.00008 |
| Transcription unit B | FBgn0014903 | 1.13 | 0.00235 |
| ***Signal transduction*** |  |  |  |
| CG32559 | FBgn0052560 | 0.68 | 0.00463 |
| CG17760 | FBgn0033756 | 0.62 | 0.00281 |
| kappaB-Ras | FBgn0040513 | 0.65 | 0.00137 |
| Lectin28C | FBgn0040099 | 0.93 | 0.00042 |
| Leucine-rich repeat 47 | FBgn0010398 | 0.59 | 0.00309 |
| Rgk2 | FBgn0085419 | 1.43 | 0.00132 |
| Rhodopsin5 | FBgn0014019 | 0.69 | 0.00197 |
| sextra | FBgn0004390 | 0.73 | 0.00323 |
| ***Apoptosis*** |  |  |  |
| Cytochrome P450 related BF6-2 | FBgn0013773 | 1.67 | 0.00259 |
| Daxx-like protein | FBgn0031820 | 0.64 | 0.00500 |
| Jun kinase | FBgn0000229 | 1.82 | 0.00044 |
| Lobe | FBgn0001332 | 0.99 | 0.00235 |
| Modifier67.2 | FBgn0002781 | 0.64 | 0.00190 |
| ***Cytochrome P450*** |  |  |  |
| Cyp6a17 | FBgn0015714 | 8.21 | 0.00027 |
| Cyp6a20 | FBgn0033980 | 2.77 | 0.00303 |
| Cyp6t1 | FBgn0031182 | 0.62 | 0.00472 |
| Cytochrome P450-4p1 | FBgn0015037 | 1.15 | 0.00215 |
| ***Aging*** |  |  |  |
| four wheel drive | FBgn0004373 | 0.87 | 0.00020 |
| Mth-like 8 | FBgn0052475 | 6.93 | 0.00130 |
| Mth-like 8 | FBgn0052475 | 7.49 | 0.00052 |
| ***Protein folding*** |  |  |  |
| DnaJ-like-60 | FBgn0020129 | 0.87 | 0.00044 |
| Heat shock protein cognate 1 | FBgn0001216 | 1.20 | 0.00250 |
| Heat shock protein cognate 1 | FBgn0001216 | 1.01 | 0.00069 |
| ***Un-grouped*** |  |  |  |
| Ance-3 | FBgn0032536 | 0.94 | 0.00308 |
| CG1017 | FBgn0035294 | 1.03 | 0.00078 |
| CG10208 | FBgn0039118 | 0.93 | 0.00220 |
| CG1146 | FBgn0035346 | 0.71 | 0.00176 |
| CG11842 | FBgn0039629 | 1.06 | 0.00141 |
| CG11880 | FBgn0039637 | 1.61 | 0.00317 |
| CG11892 | FBgn0039313 | 3.48 | 0.00124 |
| CG12111 | FBgn0030050 | 1.36 | 0.00379 |
| CG12484 | FBgn0086604 | 0.73 | 0.00062 |
| CG12885 | FBgn0039523 | 0.94 | 0.00428 |
| CG13559 | FBgn0034870 | 1.17 | 0.00324 |
| CG13568 | FBgn0034965 | 1.01 | 0.00176 |
| CG13737 | FBgn0036382 | 1.04 | 0.00117 |
| CG13822 | FBgn0039098 | 2.43 | 0.00034 |
| CG13868 | FBgn0034501 | 2.81 | 0.00058 |
| CG13897 | FBgn0035160 | 0.64 | 0.00491 |
| CG14022 | FBgn0031700 | 0.64 | 0.00092 |
| CG14075 | FBgn0036835 | 0.78 | 0.00227 |
| CG14419 | FBgn0029639 | 5.96 | 0.00404 |
| CG14481 | FBgn0034260 | 0.62 | 0.00306 |
| CG14534 | FBgn0031957 | 2.53 | 0.00493 |
| CG14535 | FBgn0031955 | 2.14 | 0.00269 |
| CG14642 | FBgn0037222 | 1.26 | 0.00334 |
| CG14643 | FBgn0037225 | 1.22 | 0.00132 |
| CG14898 | FBgn0038437 | 1.01 | 0.00245 |
| CG14984 | FBgn0035480 | 0.71 | 0.00192 |
| CG15036 | FBgn0040922 | 0.96 | 0.00166 |
| CG15046 | FBgn0030927 | 0.59 | 0.00064 |
| CG16743 | FBgn0032322 | 0.92 | 0.00002 |
| CG17325 | FBgn0040993 | 1.13 | 0.00498 |
| CG17446 | FBgn0030121 | 0.95 | 0.00113 |
| CG1824 | FBgn0030403 | 0.62 | 0.00215 |
| CG18343 | FBgn0033683 | 1.35 | 0.00374 |
| CG1941 | FBgn0033214 | 0.79 | 0.00077 |
| CG2022 | FBgn0037292 | 0.90 | 0.00021 |
| CG2162 | FBgn0035388 | 0.94 | 0.00020 |
| CG2213 | FBgn0035210 | 1.42 | 0.00080 |
| CG30038 | FBgn0050038 | 0.67 | 0.00077 |
| CG30269 | FBgn0050269 | 1.23 | 0.00204 |
| CG30273 | FBgn0050273 | 1.09 | 0.00483 |
| CG30345 | FBgn0037229 | 0.68 | 0.00077 |
| CG30424 | FBgn0050424 | 1.08 | 0.00062 |
| CG31268 | FBgn0051268 | 2.30 | 0.00118 |
| CG31445 | FBgn0051445 | 0.87 | 0.00464 |
| CG31676 | FBgn0051676 | 1.91 | 0.00256 |
| CG31778 | FBgn0051778 | 0.78 | 0.00445 |
| CG31839 | FBgn0028543 | 1.32 | 0.00229 |
| CG32267 | FBgn0052267 | 0.62 | 0.00503 |
| CG32495 | FBgn0030882 | 1.26 | 0.00098 |
| CG32500 | FBgn0052500 | 0.66 | 0.00116 |
| CG32695 | FBgn0052695 | 1.45 | 0.00181 |
| CG32944 | FBgn0052944 | 0.80 | 0.00182 |
| CG33296 | FBgn0053296 | 3.42 | 0.00062 |
| CG3397 | FBgn0037975 | 5.05 | 0.00062 |
| CG42319 | FBgn0033822 | 0.67 | 0.00301 |
| CG4364 | FBgn0032138 | 0.70 | 0.00143 |
| CG5224 | FBgn0034354 | 2.33 | 0.00409 |
| CG5316 | FBgn0038704 | 0.75 | 0.00285 |
| CG6995 | FBgn0039229 | 1.45 | 0.00255 |
| CG7054 | FBgn0038972 | 0.67 | 0.00273 |
| CG8204 | FBgn0034033 | 1.01 | 0.00457 |
| CG8745 | FBgn0036381 | 1.00 | 0.00465 |
| CG8891 | FBgn0031663 | 0.85 | 0.00364 |
| CG8920 | FBgn0027529 | 0.62 | 0.00009 |
| CG9005 | FBgn0033638 | 1.00 | 0.00181 |
| CG9028 | FBgn0036389 | 0.62 | 0.00284 |
| CG9245 | FBgn0030670 | 1.45 | 0.00251 |
| CG9273 | FBgn0032906 | 1.54 | 0.00033 |
| CG9279 | FBgn0036882 | 1.81 | 0.00159 |
| CG9451 | FBgn0036876 | 2.33 | 0.00110 |
| CG9837 | FBgn0037635 | 1.21 | 0.00443 |
| CG9989 | FBgn0039593 | 1.45 | 0.00104 |
| CHOp24 | FBgn0029709 | 0.94 | 0.00133 |
| fragment D | FBgn0015573 | 1.70 | 0.00484 |
| GUKholder | FBgn0026239 | 0.70 | 0.00156 |
| gurken | FBgn0001137 | 1.03 | 0.00112 |
| Heat shock factor | FBgn0001222 | 0.62 | 0.00014 |
| His1:CG31617 | FBgn0051617 | 0.59 | 0.00290 |
| Immune induced molecule 23 | FBgn0034328 | 5.63 | 0.00507 |
| lethal (2) essential for life | FBgn0011296 | 3.42 | 0.00381 |
| miple | FBgn0027111 | 2.72 | 0.00316 |
| MKP-like | FBgn0036369 | 1.28 | 0.00428 |
| Nup98 | FBgn0039120 | 1.06 | 0.00424 |
| Protein phosphatase N at 58A | FBgn0025573 | 0.74 | 0.00359 |
| proximal to raf | FBgn0003159 | 1.67 | 0.00096 |
| Ribosomal protein S11 | FBgn0033699 | 1.36 | 0.00297 |
| scarlet | FBgn0003515 | 0.77 | 0.00235 |
| SCP-containing protein A | FBgn0037879 | 3.16 | 0.00069 |
| SCP-containing protein C | FBgn0037879 | 3.81 | 0.00057 |
| Spn43Ad | FBgn0044011 | 0.84 | 0.00445 |
| spt4 | FBgn0028683 | 0.80 | 0.00273 |
| TBPH | FBgn0025790 | 3.42 | 0.00363 |
| totem | FBgn0035976 | 1.43 | 0.00458 |
|  |  |  |  |
| **Down-regulated genes** |  |  |  |
| ***Transcription*** |  |  |  |
| bowel | FBgn0004893 | -0.90 | 0.00091 |
| CG30080 | FBgn0050080 | -1.05 | 0.00390 |
| Complementation group C | FBgn0011656 | -2.87 | 0.00072 |
| dorsotonals | FBgn0001235 | -1.41 | 0.00004 |
| forkhead domain 64A | FBgn0004895 | -4.44 | 0.00148 |
| goliath | FBgn0004919 | -9.08 | 0.00095 |
| HDAC4 | FBgn0041210 | -0.74 | 0.00194 |
| longitudinals absent | FBgn0005630 | -2.30 | 0.00083 |
| protein kinase A | FBgn0000273 | -1.53 | 0.00340 |
| protein kinase A | FBgn0000273 | -1.39 | 0.00228 |
| quo vadis | FBgn0003396 | -2.22 | 0.00003 |
| rancor | FBgn0004656 | -1.10 | 0.00134 |
| simjang | FBgn0010762 | -2.04 | 0.00010 |
| taranis | FBgn0040071 | -1.37 | 0.00089 |
| TFIIF small subunit | FBgn0010421 | -0.92 | 0.00334 |
| ***Metabolic process*** |  |  |  |
| cap binding protein 80 | FBgn0022942 | -1.46 | 0.00416 |
| CG17322 | FBgn0027070 | -2.54 | 0.00293 |
| CG18397 | FBgn0032723 | -1.27 | 0.00341 |
| CG30359 | FBgn0050359 | -0.85 | 0.00370 |
| CG3160 | FBgn0029789 | -1.18 | 0.00073 |
| histidine decarboxylase | FBgn0005619 | -0.61 | 0.00379 |
| Phosphodiesterase 11 | FBgn0085370 | -0.64 | 0.00111 |
| Rpb4 | FBgn0053520 | -0.81 | 0.00165 |
| UDP-glycosyltransferase | FBgn0026314 | -8.91 | 0.00231 |
| UDP-glycosyltransferase 35a | FBgn0026315 | -1.25 | 0.00293 |
| ***Oxidative stress*** |  |  |  |
| CG2254 | FBgn0029994 | -2.76 | 0.00062 |
| CG31809 | FBgn0051809 | -3.86 | 0.00164 |
| CG31809 | FBgn0051809 | -3.72 | 0.00153 |
| CG4389 | FBgn0028479 | -0.95 | 0.00373 |
| CG5028 | FBgn0039358 | -0.88 | 0.00486 |
| CG9512 | FBgn0030593 | -0.69 | 0.00181 |
| DJ-1β | FBgn0039802 | -6.15 | 0.00260 |
| kekkon-like | FBgn0031016 | -1.23 | 0.00446 |
| ***Cytochrome P450*** |  |  |  |
| Cyp4ac2 | FBgn0031694 | -1.76 | 0.00001 |
| Cyp4d20 | FBgn0035344 | -0.77 | 0.00100 |
| Cyp4p2 | FBgn0033395 | -7.30 | 0.00042 |
| cytochrome P450 related AF4 | FBgn0013771 | -1.65 | 0.00119 |
| cytochrome P450-B1 | FBgn0000473 | -2.16 | 0.00131 |
| ***Signal transduction*** |  |  |  |
| CG30281 | FBgn0050281 | -1.24 | 0.00205 |
| Allatostatin Receptor 2 | FBgn0039595 | -0.86 | 0.00013 |
| Odorant receptor 49b | FBgn0028963 | -2.11 | 0.00059 |
| scabrous | FBgn0003326 | -0.67 | 0.00129 |
| ***Un-grouped*** |  |  |  |
| anon-fast-evolving-3A8 | FBgn0035236 | -0.62 | 0.00104 |
| Centrosomal and chromosomal factor | FBgn0010313 | -1.01 | 0.00500 |
| CG10112 | FBgn0033942 | -2.81 | 0.00262 |
| CG11052 | FBgn0040524 | -2.47 | 0.00426 |
| CG11123 | FBgn0033169 | -0.65 | 0.00019 |
| CG1136 | FBgn0035490 | -1.14 | 0.00133 |
| CG11779 | FBgn0038682 | -1.32 | 0.00187 |
| CG12045 | FBgn0039805 | -0.73 | 0.00198 |
| CG12179 | FBgn0025388 | -1.21 | 0.00354 |
| CG12929 | FBgn0033429 | -0.66 | 0.00431 |
| CG13004 | FBgn0030797 | -0.64 | 0.00389 |
| CG13046 | FBgn0036595 | -1.14 | 0.00234 |
| CG13130 | FBgn0040965 | -2.89 | 0.00108 |
| CG13165 | FBgn0033707 | -1.64 | 0.00432 |
| CG13222 | FBgn0033602 | -1.76 | 0.00187 |
| CG13678 | FBgn0035859 | -3.18 | 0.00140 |
| CG13705 | FBgn0035582 | -1.29 | 0.00355 |
| CG14141 | FBgn0036146 | -0.78 | 0.00059 |
| CG14224 | FBgn0031057 | -0.59 | 0.00138 |
| CG14286 | FBgn0038673 | -0.81 | 0.00250 |
| CG14352 | FBgn0031351 | -0.76 | 0.00069 |
| CG14715 | FBgn0037930 | -1.89 | 0.00126 |
| CG1702 | FBgn0031117 | -0.97 | 0.00287 |
| CG17272 | FBgn0038830 | -0.60 | 0.00220 |
| CG17350 | FBgn0032772 | -1.27 | 0.00013 |
| CG17368 | FBgn0085429 | -0.97 | 0.00151 |
| CG17739 | FBgn0033710 | -0.67 | 0.00164 |
| CG18853 | FBgn0003082 | -4.38 | 0.00002 |
| CG2157 | FBgn0030244 | -0.79 | 0.00174 |
| CG2177 | FBgn0039902 | -6.38 | 0.00033 |
| CG2915 | FBgn0033241 | -0.67 | 0.00259 |
| CG30203 | FBgn0050203 | -0.69 | 0.00424 |
| CG30382 | FBgn0026781 | -0.78 | 0.00092 |
| CG3056 | FBgn0024987 | -1.33 | 0.00194 |
| CG31344 | FBgn0051344 | -0.79 | 0.00128 |
| CG32027 | FBgn0052027 | -2.19 | 0.00298 |
| CG32132 | FBgn0052132 | -1.61 | 0.00106 |
| CG32412 | FBgn0052412 | -0.67 | 0.00339 |
| CG32572 | FBgn0052572 | -3.46 | 0.00112 |
| CG32822 | FBgn0052822 | -1.97 | 0.00479 |
| CG32850 | FBgn0052850 | -2.16 | 0.00350 |
| CG33329 | FBgn0053329 | -0.59 | 0.00137 |
| CG3552 | FBgn0035999 | -0.68 | 0.00246 |
| CG3604 | FBgn0031562 | -1.79 | 0.00058 |
| CG3822 | FBgn0038837 | -2.44 | 0.00085 |
| CG3837 | FBgn0038279 | -0.65 | 0.00079 |
| CG3857 | FBgn0023520 | -1.23 | 0.00091 |
| CG3984 | FBgn0038291 | -1.69 | 0.00243 |
| CG40191 | FBgn0058191 | -0.69 | 0.00285 |
| CG42362 | FBgn0034648 | -0.68 | 0.00362 |
| CG4377 | FBgn0034664 | -0.66 | 0.00049 |
| CG4630 | FBgn0033809 | -1.36 | 0.00117 |
| CG5023 | FBgn0038774 | -0.73 | 0.00401 |
| CG5335 | FBgn0034365 | -0.82 | 0.00204 |
| CG5741 | FBgn0035937 | -1.66 | 0.00155 |
| CG6293 | FBgn0037807 | -2.12 | 0.00198 |
| CG6912 | FBgn0038290 | -5.14 | 0.00004 |
| CG7224 | FBgn0031971 | -0.60 | 0.00164 |
| CG7529 | FBgn0037090 | -1.46 | 0.00175 |
| CG7607 | FBgn0036145 | -0.73 | 0.00157 |
| CG7694 | FBgn0038627 | -0.77 | 0.00072 |
| CG7770 | FBgn0036918 | -0.64 | 0.00242 |
| CG7900 | FBgn0037548 | -3.00 | 0.00039 |
| CG8533 | FBgn0036907 | -1.65 | 0.00233 |
| CG8979 | FBgn0033669 | -0.80 | 0.00408 |
| CG9080 | FBgn0033593 | -0.83 | 0.00250 |
| CG9589 | FBgn0038361 | -7.23 | 0.00374 |
| CG9865 | FBgn0034649 | -0.65 | 0.00201 |
| dpr15 | FBgn0037993 | -5.84 | 0.00085 |
| dpr8 | FBgn0052600 | -2.00 | 0.00313 |
| enabled | FBgn0000578 | -1.62 | 0.00473 |
| enhanced adult sensory threshold | FBgn0010110 | -0.76 | 0.00194 |
| Eukaryotic initiation factor 4B | FBgn0020660 | -1.53 | 0.00140 |
| gag-int-pol | FBgn0013437 | -1.39 | 0.00242 |
| Inscutable | FBgn0011674 | -1.11 | 0.00241 |
| Kinesin-like protein at 98A | FBgn0004387 | -0.75 | 0.00097 |
| mitochondrial ribosomal protein L39 | FBgn0036462 | -0.81 | 0.00225 |
| mitochondrial ribosomal protein S28 | FBgn0034361 | -1.13 | 0.00036 |
| m-spondin | FBgn0020269 | -0.70 | 0.00070 |
| neuroglian | FBgn0002968 | -0.67 | 0.00177 |
| N-methyl-D-aspartate receptor-associated protein | FBgn0013305 | -0.68 | 0.00223 |
| Odorant-binding protein 56g | FBgn0034474 | -0.80 | 0.00025 |
| Organic anion transporting polypeptide 30B | FBgn0032123 | -1.90 | 0.00049 |
| photolyase | FBgn0003082 | -3.82 | 0.00028 |
| Prosap | FBgn0040752 | -1.93 | 0.00072 |
| Ranbp9 | FBgn0037894 | -0.74 | 0.00356 |
| Ribonuclear protein at 97D | FBgn0004903 | -1.08 | 0.00437 |
| Ribosomal protein L24 | FBgn0032518 | -0.97 | 0.00408 |
| SCAP | FBgn0033052 | -2.76 | 0.00012 |
| Sno-oncogene | FBgn0085450 | -2.27 | 0.00378 |
| trailer hitch | FBgn0041775 | -0.71 | 0.00086 |
| transformer-2 | FBgn0003742 | -0.97 | 0.00368 |
| Trim9 | FBgn0051721 | -0.85 | 0.00502 |
| vasa intronic gene | FBgn0024183 | -1.05 | 0.00178 |
|  |  |  |  |
